# A role for alternative splicing in circadian control of exocytosis and glucose homeostasis

**DOI:** 10.1101/gad.338178.120

**Published:** 2020-08-01

**Authors:** Biliana Marcheva, Mark Perelis, Benjamin J. Weidemann, Akihiko Taguchi, Haopeng Lin, Chiaki Omura, Yumiko Kobayashi, Marsha V. Newman, Eugene J. Wyatt, Elizabeth M. McNally, Jocelyn E. Manning Fox, Heekyung Hong, Archana Shankar, Emily C. Wheeler, Kathryn Moynihan Ramsey, Patrick E. MacDonald, Gene W. Yeo, Joseph Bass

**Affiliations:** 1 Department of Medicine, Division of Endocrinology, Metabolism, and Molecular Medicine, Northwestern University Feinberg School of Medicine, Chicago, Illinois 60611, USA;; 2 Department of Cellular and Molecular Medicine, University of California at San Diego, La Jolla, California 92093, USA;; 3 Department of Pharmacology, Alberta Diabetes Institute, University of Alberta, Edmonton, Alberta T6G 2E1, Canada;; 4 Center for Genetic Medicine, Northwestern University, Chicago, Illinois 60611, USA

**Keywords:** circadian clock, insulin secretion, exocytosis, alternative splicing, RNA sequencing, transcriptomics, THRAP3, MADD, CASK, SNAP25

## Abstract

The circadian clock is encoded by a negative transcriptional feedback loop that coordinates physiology and behavior through molecular programs that remain incompletely understood. Here, we reveal rhythmic genome-wide alternative splicing (AS) of pre-mRNAs encoding regulators of peptidergic secretion within pancreatic β cells that are perturbed in *Clock*^−/−^ and *Bmal1*^−/−^ β-cell lines. We show that the RNA-binding protein THRAP3 (thyroid hormone receptor-associated protein 3) regulates circadian clock-dependent AS by binding to exons at coding sequences flanking exons that are more frequently skipped in clock mutant β cells, including transcripts encoding *Cask* (*calcium/calmodulin-dependent serine protein kinase*) and *Madd* (*MAP kinase-activating death domain*). Depletion of THRAP3 restores expression of the long isoforms of *Cask* and *Madd*, and mimicking exon skipping in these transcripts through antisense oligonucleotide delivery in wild-type islets reduces glucose-stimulated insulin secretion. Finally, we identify shared networks of alternatively spliced exocytic genes from islets of rodent models of diet-induced obesity that significantly overlap with clock mutants. Our results establish a role for pre-mRNA alternative splicing in β-cell function across the sleep/wake cycle.

## Abstract (precis)

In this study from Marcheva et al., the authors show that rhythmic genome-wide alternative splicing (AS) of pre-mRNAs encoding regulators of peptidergic secretion within pancreatic β cells is perturbed in Clock^−/−^ and Bmal1^−/−^ β-cell lines. They find that RNA-binding protein THRAP3 regulates circadian clock-dependent AS by binding to exons at coding sequences flanking exons that are more frequently skipped in clock mutant β cells, including transcripts encoding Cask and Madd, thus establishing a role for the temporal control of pre-mRNA alternative splicing in the secretory dynamics of mammalian β cells throughout the sleep/wake cycle.

The circadian clock is an endogenous timing system that coordinates organismal physiology in anticipation of the sleep–wake cycle (Bass and Takahashi 2010; Asher and Schibler 2011; Bass 2012). At the molecular level, the clock is composed of basic helix–loop–helix transcription factors (TFs), in which activators in the forward limb (CLOCK and BMAL1) induce the transcription of their own repressors (PER1/2/3 and CRY1/2) in a cycle that repeats itself every 24 h (Balsalobre et al. 1998; Preitner et al. 2002; Liu et al. 2008; Bugge et al. 2012; Cho et al. 2012). The REV-ERB and ROR proteins form a reinforcing loop that modulates *Bmal1* transcription. In turn, the core TFs induce robust amplitude in the rhythmic production of downstream PAR-bZIP TFs in liver and β cells that play key roles in mammalian metabolism (Gachon et al. 2006; Tong et al. 2010; Nakabayashi et al. 2013). RNA sequencing analyses have shown that CLOCK/BMAL1 drive rhythmic expression of 10%–25% of all mRNAs within diverse mammalian tissues (Panda et al. 2002; Lowrey and Takahashi 2004; Hughes et al. 2009; Vollmers et al. 2009). Unique sets of genes oscillate across tissues due to interactions between CLOCK/BMAL1 and lineage-determining factors (Lee et al. 2005; Perelis et al. 2015). Surprisingly, however, recent reports in liver have indicated that ∼70% of genes that display mRNA rhythmicity do not display oscillations in their corresponding intron-containing pre-mRNAs (Koike et al. 2012). Additionally, the zenith of CLOCK/BMAL1 chromatin binding does not correspond to the peak phase of mRNA accumulation for most target genes. These studies suggest that posttranscriptional regulation is a crucial component of circadian gene regulation (Koike et al. 2012; Menet et al. 2012; Green 2018).

Mounting evidence suggests that posttranscriptional RNA-processing events such as methylation, polyadenylation, and alternative splicing (AS) are linked to the circadian clock (Kojima et al. 2012; McGlincy et al. 2012; Fustin et al. 2013). AS, which enables a single genomic locus to produce multiple functionally distinct mRNAs in a tissue-, cell type-, and developmental stage-specific manner (Young et al. 1981; Vuong et al. 2016; Kim et al. 2018), is initiated upon the recruitment of RNA-binding proteins (RBPs) to consensus RNA motifs within regulatory elements of introns and exons of pre-mRNAs. RBPs then guide the oligomeric spliceosome complex to the correct sites for subsequent inclusion, exclusion, or skipping of exon cassettes to generate alternatively spliced transcripts. Rhythmic production of unique alternative mRNA splice isoforms from pre-mRNA has been shown to occur in plants, *Drosophila*, and mice (Sanchez et al. 2010; Hughes et al. 2012; McGlincy et al. 2012; Wang et al. 2018). Oscillating AS events have also been shown to be driven by both the internal core circadian clock and environmental timing cues such as temperature, which drives the rhythmic phosphorylation and function of serine–arginine (SR)-containing spliceosomal proteins as well as the splicing efficiency and stabilization of *cold-inducible RNA-binding protein* (*Cirp*) (Morf et al. 2012; Gotic et al. 2016; Preußner et al. 2017).

Evidence suggests that the clock regulates AS in neurons, where neurotransmitter release varies across the day/night cycle (Shinohara et al. 2002; Barber et al. 2016; Gizowski et al. 2016; Wang et al. 2018), raising the possibility that rhythmic AS may play a role in physiology. Genetic studies show that selective ablation of the molecular clock within pancreatic β cells of adult mice induces diabetes due to severely impaired insulin secretion (Marcheva et al. 2010; Sadacca et al. 2011; Perelis et al. 2015; Petrenko et al. 2017). Clues to a role for rhythmic AS in β-cell function originated in RNA and ChIP sequencing studies in mouse and human pancreatic islets, which revealed that as many as 80% of rhythmic mRNAs and 82% of mRNAs differentially expressed in *Bmal1*^−/−^ islets are not directly controlled by CLOCK/BMAL1, and are therefore regulated either transcriptionally or posttranscriptionally (Perelis et al. 2015).

Since the circadian transcriptome in islet cells is enriched for mRNAs encoding synapse-associated proteins that are established targets of AS, we sought here to test whether AS events are regulated by the clock through circadian RBP expression in islet cells. We used genome-wide transcriptomics to quantify changes in AS events and expression in circadian controlled RNAs. We found that wild-type (WT) islets display rhythmic expression of RBPs and AS of mRNAs encoding protein regulators of peptidergic secretion, and that clock nullizygous β cells display significantly increased exon skipping events in genes that control insulin secretion. We report a novel role for the circadian RBP THRAP3 in isoform selection within misspliced transcripts associated with insulin secretion in clock-deficient cells. In addition, we used live cell capacitance measurements to determine the level of impairment in exocytosis in new CRISPR-generated clock mutant β-cell lines as well as primary β cells from pancreas-specific *Bmal1*^−/−^ mice. Last, we identify an overlap between AS events that are disrupted in clock mutant β cells and those identified in diet-induced obesity. Thus, we propose that RNA missplicing in β cells contributes to the etiology of diabetes mellitus in conditions of circadian disruption.

## Results

### Circadian control of alternative splicing in pancreatic islets throughout the day

**Figure 1.**
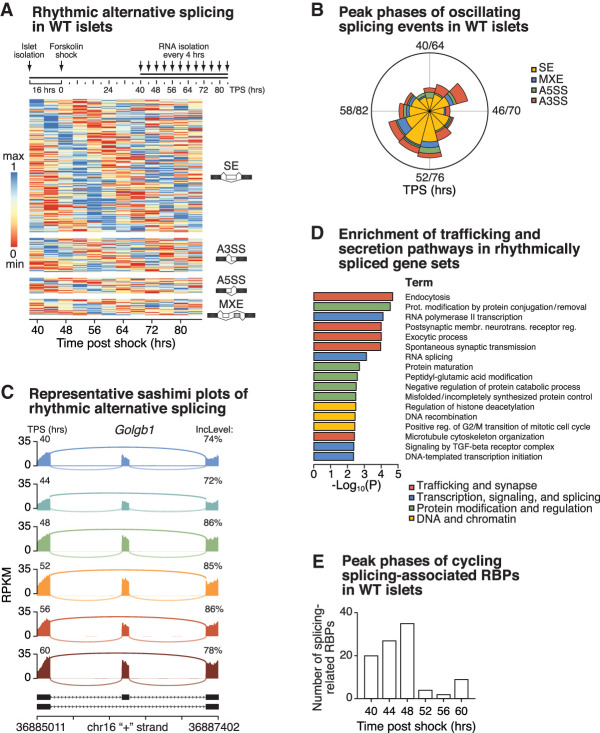
Circadian control of alternative splicing in pancreatic islets throughout the day. (*A*) Schematic of ex vivo experimental design for RNA isolation in forskolin-synchronized mouse islets. RNA was isolated every 4 h for 48 h starting 40 h after forskolin shock. TPS (hours), time after shock (hours) (*n* = 3/timepoint). Heatmap representing rhythmically spliced genes (skipped exons [SE], alternative 3′ and 5′ splice sites [A3SS and A5SS], and mutually exclusive exons [MXE]) every 4 h over the course of 48 h in forskolin-synchronized oscillating WT islets. (*B*) Radial histogram showing the number of splicing events of each type with peak psi occurring within each period length-adjusted phase, with the radius corresponding to 40 splicing events. (*C*) Representative sashimi plots show time of day-dependent alternative splicing of *Golgb1* every 4 h across 24 h (starting 40 h after forskolin shock), with the exon inclusion level indicated at each time point. (*D*) Pathway analyses reveal significant enrichment of trafficking and secretion pathways within alternatively spliced gene sets. (*E*) Peak phase in expression of cycling RNA-binding proteins within the GO ontology term “RNA splicing.”

To determine whether posttranscriptional alternative pre-mRNA splicing in pancreatic islets is influenced by circadian time, we first assessed genome-wide AS events from WT mouse islets cultured ex vivo and sampled in triplicate at 4-h intervals for 48 h following synchronization by a 1-h 10 μM forskolin shock (Perelis et al. 2015) (GSE69889) (see schematic in Fig. 1A). We used rMATS (replicate multivariate analysis of transcript splicing) (Shen et al. 2014) to identify and quantify the following types of AS events: skipped exons (SE), alternative 3′ and 5′ splice sites (A3SS and A5SS), and mutually exclusive exons (MXE) (Fig. 1A). To compare AS events across time, we first collected RNA sequencing (RNA-seq) reads in our data that uniquely aligned to exon–exon junctions annotated within the reference genome that were commonly assigned as AS events in all time points that had changes in percent-spliced in (ψ) values reflecting exon inclusion in at least half of all averaged time points. The ensemble of AS events in our data included 11,400 SEs, 2251 A3SSs, 1297 A5SSs, and 1689 MXEs across 6520 unique genes. To establish the trajectories of AS events throughout the time series to assess rhythmic AS across the day, we calculated ψ for all common AS events from each of the three replicate samples for each time point and identified statistically significant (adjusted *P*-value < 0.05) oscillations in ψ using JTK_cycle (Hughes et al. 2010), allowing for period lengths of 20, 24, or 28 h. This analysis revealed a total of 257 oscillatory AS events, consisting of 172 SE, 44 A3SS, 20 A5SS, and 21 MXE events across 241 unique genes (Fig. 1A; Supplemental Table S1), which collectively displayed a bimodal distribution in peak phases occurring ∼44 and 52 h after shock (Fig. 1B). Sashimi plots, which show reads mapping across splice junctions to quantify exon inclusion levels, are shown across the circadian day to demonstrate rhythmic AS for *Golgb1*, which encodes a Golgi-associated and vesicle tethering factor (Fig. 1C; Supplemental Fig. S1A). Rhythmic AS for other representative genes is shown by plotting inclusion level differences for cassette exons across the circadian day in the splicing factor *Srrm2* and the synaptic vesicle cycle-associated GTPase *Dnm1* (Supplemental Fig. S1A). These data establish for the first time that the molecular clock regulates rhythmic AS throughout the day in the pancreatic islet, a peripheral cell type.

Given that the islet circadian transcriptome is enriched for mRNAs encoding trafficking and synapse-associated proteins known to be regulated by AS in neuronal tissues, we hypothesized that similar pathways might emerge within circadian-controlled alternatively spliced transcripts. Indeed, interrogation of functional pathways included in KEGG (Kyoto Encyclopedia of Genes and Genomes), GO (gene ontology), and reactome databases using Metascape (Tripathi et al. 2015) revealed that genes with oscillatory splicing events are over-represented in trafficking and synapse pathways (Fig. 1D), including endocytosis (i.e., *Dnm1*, *Pip5k1a*, *Vps25*, *Rab11a*, and *Smap2*), spontaneous synaptic transmission (i.e., *Rims2*, *Mtmr2*, and *App*), and exocytic processes (i.e., *Grik5*, *Snap47*, *Vps11*, and *Synj1*). Several pathways and gene targets regulating transcription and posttranscriptional processing were also identified (Fig. 1D), including the transcript for splicing factor SRRM2 (Supplemental Fig. S1A). Interestingly, comparison of rhythmically spliced transcripts (from Fig. 1A) and rhythmically expressed transcripts (from Perelis et al. 2015) revealed that while only 34% of rhythmically spliced transcripts overlapped with rhythmically expressed mRNAs, both were enriched for gene sets important for synaptic and secretory pathways (Supplemental Fig. S1B).

Since AS occurs as a consequence of interactions between RBPs and splicing regulatory elements in pre-mRNAs, we next interrogated the islet transcriptome for rhythmically expressed RBPs and identified 296 oscillating transcripts encoding RBPs (Supplemental Fig. S1C; Supplemental Table S2). We identified oscillation of RBPs such as *Nono* and *Adarb1*, which have been implicated in regulating circadian RNA transport and editing, respectively (Terajima et al. 2017; Benegiamo et al. 2018). Of note, of the 296 rhythmic RBPs, 99 have shown to been associated with splicing (Fig. 1E; Supplemental Table S2), including *Thrap3* (Lande-Diner et al. 2013). We further observed that rhythmic RBPs, including the splicing-associated RBPs, displayed peak phases of expression ∼44–48 h after shock (Fig. 1E; Supplemental Fig. S1C), which overlapped with the first peak in splicing (Fig. 1B) and the zenith in insulin secretion (Perelis et al. 2015). Conversely, the trough of RBP expression (Fig. 1E; Supplemental Fig. S1C) coincided with the second peak in splicing ∼52–56 h after shock (Fig. 1B) and was in anticipation of the nadir in insulin secretion (Perelis et al. 2015).

### Generation of cell models for analysis of BMAL1 and CLOCK alternative splicing and function

Given that alternative splicing of mRNAs encoding key exocytotic and trafficking factors varies across the day in islet cell clusters (Fig. 1D), and that both clock-controlled expression and AS are cell type-specific (Young et al. 1981; Vuong et al. 2016), we next sought to determine the specific role of the β-cell molecular circadian clock in regulating the synthesis and processing of protein-coding messenger RNAs. To do so, we first generated clonal isogenic β-cell lines lacking a functional clock (*Bmal1*^−/−^ or *Clock*^−/−^) that recapitulate physiologic features of glucose-responsive insulin secretion. We used CRISPR/Cas9 gene editing and homology-directed repair (HDR) in mouse β-TC6 cells to establish *Bmal1* and *Clock* nullizygous β cells harboring premature stop codons that lead to disruption of exons encoding the bHLH DNA-binding domains (Supplemental Fig. S2A). Quantitative real-time PCR and Western blot screening of individual clones identified desired cell lines lacking functional *Bmal1* and *Clock* mRNA and protein, as well as decreased expression of their downstream target *Rev-erbα* (Supplemental Fig. S2B,C). To confirm functional loss of the β-cell core molecular clock, we transduced *Bmal1*^−/−^, *Clock*^−/−^, and control β-cell lines with a lentivirus encoding a *Per2* promoter fragment containing consensus binding sites for core circadian TFs immediately upstream of the firefly *Luciferase* (*Luc*) gene that is sufficient to drive circadian bioluminescence rhythms in mammalian cells (Supplemental Fig. S2D; Liu et al. 2008). Whereas control β-cell lines transduced with the *Per2*-*dLuc* reporter displayed robust circadian bioluminescence rhythms following synchronization by exposure to a 24-h temperature cycle that mimics the endogenous body temperature rhythm (Buhr et al. 2010; Saini et al. 2012), PER2-dLUC failed to oscillate in *Bmal1*^−/−^ and *Clock*^−/−^ β cells, indicating a loss of the core circadian network function in these cells (Supplemental Fig. S2D). Consistent with β-cell-specific loss of clock function, these *Bmal1*^−/−^ and *Clock*^−/−^ β-cell lines had impaired nutrient and nonnutrient secretagogue-induced insulin secretion following exposure to either glucose or potassium chloride, respectively (Supplemental Fig. S2E; Marcheva et al. 2010; Perelis et al. 2015). Importantly, these *Bmal1*^−/−^ and *Clock*^−/−^ β-cell lines have secretory phenotypes that are shared with pancreas-specific *Bmal1* mutant mice (Marcheva et al. 2010; Sadacca et al. 2011; Perelis et al. 2015). Here, we also show a similar loss of glucose-responsive insulin secretion in pancreas-specific *Clock* knockout mice. Ablation of CLOCK expression specifically in pancreatic islets was confirmed by immunofluorescent staining (Supplemental Fig. S3A) and did not alter locomotor activity, circadian period length, feeding rhythm, or body weight (Supplemental Fig. S4). Similar to pancreas-specific loss of BMAL1, we observed significant hyperglycemia without a concomitant increase in insulin (Supplemental Fig. S3B,C), and *PdxCre;Clock^flx/flx^* mice were glucose intolerant and displayed reduced insulin secretion following a glucose challenge compared with their littermate controls (Supplemental Fig. S3D). Finally, islets isolated from pancreas-specific *Clock* KO mice also secreted significantly less insulin in response to both glucose and KCl, an insulin secretagogue that causes direct β-cell depolarization, despite no change in intracellular insulin content, consistent with observations in CRISPR cell lines (Supplemental Fig. S3E). Together, these findings in vivo and in cell lines are the first to show that both BMAL1 and CLOCK TFs are required for appropriate control of stimulus-secretion coupling within β cells and prompted us to interrogate genome-wide transcription and processing of mRNAs underlying the circadian control of insulin secretion in pancreatic β cells versus in a heterogeneous islet population. Our results demonstrate a cell-autonomous requirement for molecular clock components in β-cell function.

### BMAL1 and CLOCK control shared exocytic networks regulated by alternative splicing

**Figure 2.**
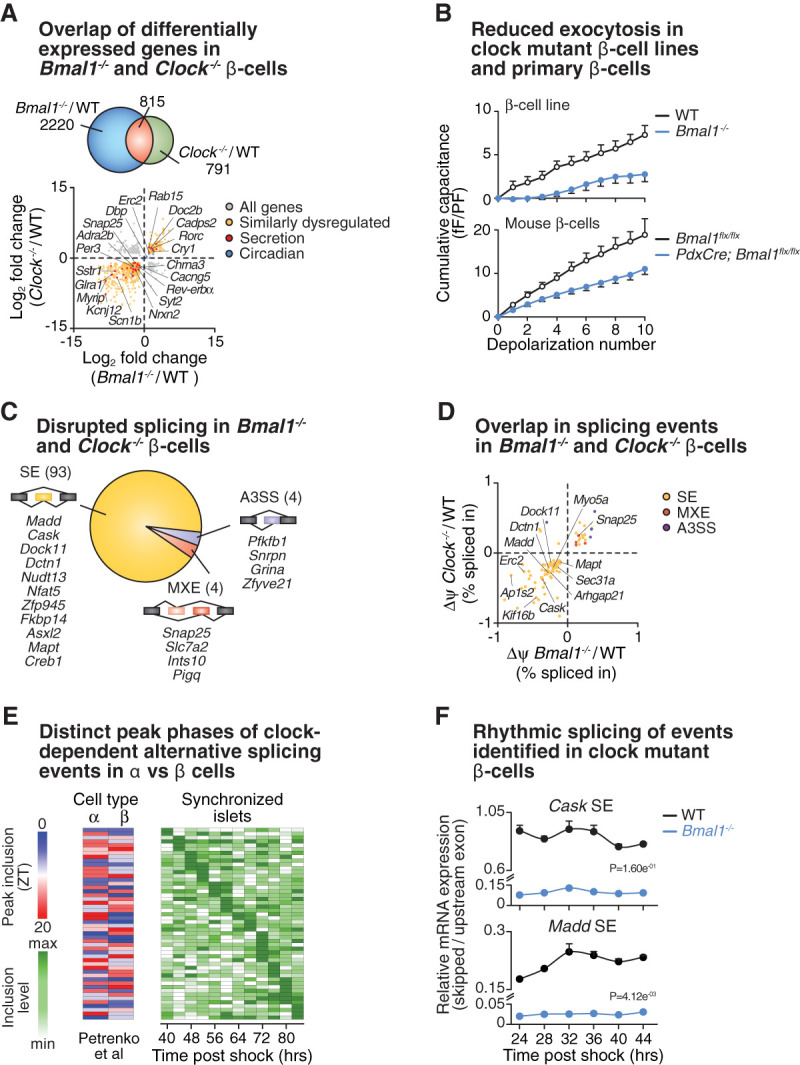
Ablation of both BMAL1 and CLOCK disrupts splicing within gene networks important in vesicle trafficking and exocytosis. (*A*, *top*) Venn diagram of overlapping differentially expressed genes identified by RNA sequencing in *Bmal1*^−/−^ and *Clock*^−/−^ β-cell lines compared with control cell line. (*Bottom*) Scatter plot depicts all differentially expressed genes, highlighting secretion and circadian genes with a strong correlation between directionality of gene expression changes between *Bmal1*^−/−^ and *Clock*^−/−^ β-cell lines. (*B*) Capacitance measurements in *Bmal1*^−/−^ β-cell lines (*n* = 13–17 cells per genotype) and β cells from *PdxCre;Bmal1^flx/flx^* mouse islets (*n* = 4–5 mice per genotype, 7–16 cells per mouse) compared with controls. (*C*) Splicing analysis reveals differential alternative splicing events (skipped exons [SE], mutually exclusive exons [MXE], and alternative 3′ splice sites [A3SS]) common in both *Bmal1*^−/−^ and *Clock*^−/−^ β-cell lines. Representative genes are listed *below* each category. (*D*) Scatter plot depicts alternatively spliced genes common to both *Bmal1*^−/−^ and *Clock*^−/−^ β-cell lines, highlighting strong correlation of directionality between *Bmal1*^−/−^ and *Clock*^−/−^ β-cell lines. (*E*) Zeitgeber time (ZT) of peak inclusion in sorted α and β cells from islets (*left*) (Petrenko et al. 2017) and patterns of mean ψ scores across two 24-h timescales for skipped exon events identified in clock mutants in synchronized islets (*right*). (*F*) Rhythmic splicing events identified via qPCR in forskolin-synchronized WT and *Bmal1*^−/−^ β-cell lines (*n* = 3/timepoint). RNA was isolated every 4 h across 24 h (starting 24 h after forskolin shock). Skipped exon expression was normalized to a neighboring (nonspliced) exon, and evaluation for rhythmicity performed by JTK_CYCLE (adjusted *P*-value shown).

To first identify the repertoire of genes specifically regulated by both BMAL1 and CLOCK in β cells and to ultimately determine whether circadian regulation of RNA processing may also contribute to secretory defects in clock mutant β cells, we initially performed RNA sequencing (RNA-seq) comparing polyadenylated mRNAs isolated from *Bmal1*^−/−^, *Clock*^−/−^, and WT control β cells. We identified altered expression of 3035 genes in *Bmal1*^−/−^ cells and 1606 genes in *Clock*^−/−^ cells (log_2_ FC > 1, FDR < 0.01) (Fig. 2A). Among these differentially expressed genes, we focused on the 815 mRNAs that were differentially expressed in both *Clock*^−/−^ and *Bmal1*^−/−^ cells, as they are more likely to underlie the common secretory phenotypes observed in these two lines (Supplemental Table S3). Importantly, 79% (640/815) of the commonly differentially expressed genes displayed common directionality (up-regulated or down-regulated in both), including a large number of genes related to secretory processes (Fig. 2A), suggesting a high degree of overlap of target genes regulated by both core clock activators, as opposed to being CLOCK or BMAL1 targets independent of the core clock network. Among these commonly differentially expressed mRNAs, 21% (136/640) were up-regulated while 79% (504/640) were down-regulated, consistent with inhibition of a transcription activation complex (Fig. 2A; Supplemental Fig. S5A). To identify the functional β-cell gene networks regulated by both CLOCK and BMAL1, we tested for over-representation of defined KEGG, GO, and reactome pathways amongst the genes that were commonly changed in both CLOCK and BMAL1 mutant cell lines using Metascape (Tripathi et al. 2015). Consistent with inhibition of insulin secretion, the 504 down-regulated mRNAs common to both CLOCK and BMAL1 knockout cells were enriched for genes with annotated functions related to G-protein and ion channel activation, synaptic processes, and regulation of hormone secretion (Supplemental Fig. S5B). Among these were neurotransmitter and hormone receptors mediating responses to somatostatin (*Sstr1*, *Sstr3*, and *Sstr5*), acetylcholine (*Chrna3* and *Chrna4*), norepinephrine (*Adra2b*), dopamine (*Drd2*), glycine (*Glra1*), and glutamate (*Gria4*), as well as voltage-gated calcium (*Cacng5* and *Cacna2d4*), sodium (*Scn1b*), and potassium channels (*Kcne1*, *Kcnh3*, *Kcnj12*, *Kcnk1*, *Kcnma1*, and *Kcnq2*). mRNAs encoding proteins mediating the synthesis or downstream effectors of second messenger signals initiated by membrane-bound receptors such as calcium, 3′,5′ cyclic adenosine monophosphate (cAMP), and diacyl glycerol were also inhibited in clock mutant cells (*Syt2*, *Syt16, Adcy5*, *Rgs4*, and *Dgkb*). Last, clock mutant cells displayed altered expression of mRNAs encoding proteins regulating organelle localization (*Rab15* and *Myrip*) and synaptic vesicle priming and exocytosis (*Doc2b* and *Cadps2*). These changes in the transcriptomes of CLOCK- and BMAL1-deficient cells suggest altered expression and function of proteins coupling nutrient sensing to the regulated release of insulin. Indeed, electrophysiologic analyses revealed that *Bmal1* mutant β-cell lines and primary mouse β cells from *PdxCre;Bmal1^flx/flx^* mice display reduced rates of exocytosis following direct depolarization as indicated by reduced capacitance in the circadian mutant β-cell lines and mouse β cells (Fig. 2B). The set of commonly differentially expressed genes also included mRNAs encoding synaptic membrane-associated proteins that are functionally regulated by AS in neurons (*Snap25*, *Nrxn2*, and *Erc2*), which prompted us to next interrogate the impact of disruption of the molecular clock on posttranscriptional pre-mRNA AS in β cells.

Given that synchronized WT islets display rhythmic AS (Fig. 1) and that clock control of peptide secretion in pancreatic β cells is similar to that in neurons, we next assessed genome-wide AS patterns in *Clock* and *Bmal1* mutant β cells. We hypothesized that circadian control of RNA processing may also contribute to cell type-specific circadian gene regulation and insulin secretion in the β cell. We used rMATS to quantify AS events in *Clock* and *Bmal1* mutant cell lines relative to WT. Considering both intron spanning and exonic reads, we detected 1453 AS events in *Bmal1* mutant cells (FDR < 0.05, ψ > 0.1) consisting of 1018 skipped exons (SE), 174 mutually exclusive exons (MXE), 134 alternative 3′ splice sites (A3SS), and 127 alternative 5′ splice sites (A5SS). In *Clock* mutant cells, we detected 710 AS events, of which 492 were SE, 105 were MXE, 63 were A3SS, and 50 were A5SS events. Applying the same filtering approach as for mRNA expression, we identified 101 AS events within 95 annotated genes that were common and displayed similar directionality in both *Bmal1*^−/−^ and *Clock*^−/−^ cells (Fig. 2C,D; Supplemental Table S4). A majority of the common AS events were SE (93), with the remaining as MXE (four) and A3SS (four) events (Fig. 2C). Only four of these 95 alternatively spliced genes were included among the 640 differentially expressed mRNAs (Supplemental Fig. S5D), suggesting that changes in AS and mRNA abundance are regulated by distinct mechanisms downstream from the molecular clock. Differentially spliced mRNAs encoded proteins known to mediate cellular events important for insulin vesicle transport and exocytosis, such as cytoskeletal and motor proteins (*Mapt*, *Arhgap21*, *Myo5a*, *Kif16b*, and *Dctn1*), vesicle membrane constituents (*Erc2*, *Ap1s2*, and *Sec31a*), synaptic vesicle-associated proteins (*Snap25*), and kinases and GTPases responsible for posttranslational modifications to the secretory apparatus (*Cask* and *Madd*) (Fig. 2D; Supplemental Fig. S5C).

Previous studies have used FACS-sorting to uncover distinct circadian transcriptional profiles of α and β cells, revealing that exocytic genes in these two cell populations within the islet oscillate in distinct phases (Petrenko et al. 2017). To specifically test whether α- and β-cell types display differences in peaks of AS events across the day, we examined rhythmic patterns of AS events in FACS-sorted α and β cells collected every 4 h across a 24-h circadian cycle by performing rMATS analyses on this published data set (GSE95156), as we did with the synchronized islets in Figure 1A. Indeed, we found that a majority of the transcripts that displayed altered splicing in the clock-deficient β cells (Fig. 2D), but that were not identified as statistically rhythmic in whole islets, exhibited peak exon inclusion at distinct times of day in α versus β cells (Fig. 2E). To independently determine whether the differentially spliced transcripts identified in the clock mutant β-cell lines (Fig. 2D) displayed rhythmicity in a pure WT β-cell population, we synchronized WT β-TC6 cells (Supplemental Fig. S5E) and performed targeted qPCR analysis of representative differentially spliced (SE) transcripts, including the secretion-related genes *Cask*, *Madd*, *Dock11*, and *Dctn1*. We observed patterns of rhythmic exon inclusion in these key targets, which exhibit high degrees of AS under control of CLOCK/BMAL1 (Fig. 2F; Supplemental Fig. S5F), indicating that splicing events that are perturbed by clock mutation display rhythms in a pure population of β cells. Together these data reveal a connection between the activating limb circadian TFs and rhythmic splicing in pancreatic β cells.

### Thyroid hormone receptor associated protein 3 (THRAP3) regulates circadian clock-dependent alternative splicing

**Figure 3.**
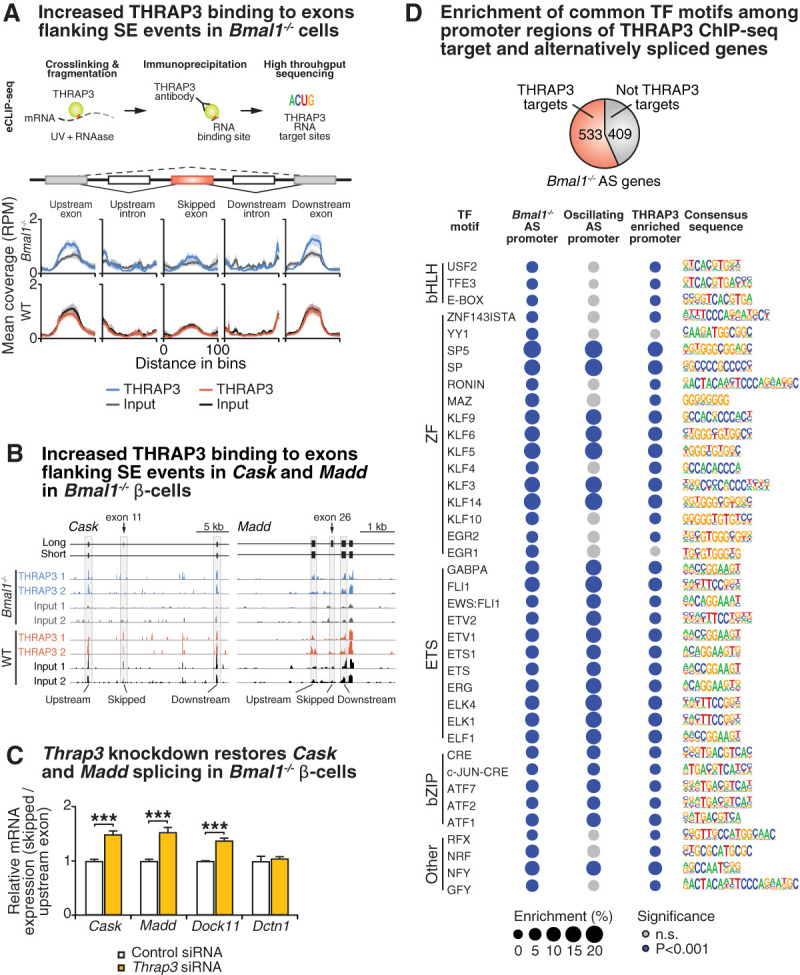
THRAP3 mediates clock-dependent alternative splicing. (*A*) Schematic of eCLIP sequencing experiment (*top*) and normalized (reads per million) THRAP3 eCLIP or input sequencing reads along indicated regions of alternatively spliced mRNAs (*bottom*). Solid line indicates mean for two replicates and shaded area represents 95% confidence interval. (*B*) UCSC genome browser tracks showing reads from indicated THRAP3 IP and input samples along regions within *Cask* and *Madd* RefSeq transcripts. Skipped exons identified by rMATS and flanking exons are shaded in gray and track heights for each sample are standardized to equal height for row shown. (*C*) *Thrap3* siRNA knockdown restores exon inclusion in *Bmal1*^−/−^ β cells, assessed by qPCR (*n* = 3). (*D*, *top*) Pie chart summarizing number of AS genes in *Bmal1*^−/−^ β cells identified as THRAP3 ChIP-seq targets. (*Bottom*) Bubble plot indicating enrichment of indicated motifs within promoter regions of AS genes and promoter-localized THRAP3 peaks. Size of bubbles reflects enrichment score for motifs within each indicated set of promoters, and those called as statistically significant (Homer hypergeometric test *P* < 0.05) are shown in blue.

Given our observation that rhythmic splicing occurs under the control of core circadian transcription factors (TFs), we next sought to examine how the clock TFs regulate AS in pancreatic β cells by examining RBPs displaying rhythmic expression in islet cells (Supplemental Table S2). Identification of rhythmic expression of the gene encoding the RBP THRAP3 (thyroid hormone receptor-associated protein 3) (Supplemental Fig. S6A ) was of particular interest since THRAP3 has been shown to regulate AS by interacting with splicing factors (Nakatsuru et al. 1989; Yarosh et al. 2015). It also binds CLOCK/BMAL1 to regulate rhythmic transcription (Lande-Diner et al. 2013) and metabolic TFs such as PPARγ recruit THRAP3 to chromatin (Choi et al. 2014). To determine whether THRAP3 binds to differentially spliced transcripts in clock mutant β-cell lines, we performed enhanced UV cross-linking and immunoprecipitation (eCLIP), followed by sequencing analysis (Van Nostrand et al. 2016) in two replicate WT and *Bmal1*^−/−^ lysates. Briefly, THRAP3–RNA complexes were immunoprecipitated, and ribonucleoprotein (RNP) complexes within the expected size range were isolated following electrophoresis and transfer to nitrocellulose membranes. For each IP replicate, a corresponding input lysate was processed to isolate a size-matched input RNA sample, comprising RNAs within the background set of RNPs similarly sized to THRAP3 complexes. We note that size matching to input is necessary in order to control for differences in RNA expression levels across the genome. Illumina sequencing libraries were then generated from both WT and *Bmal1*^−/−^ β-cell-derived IP and input RNA (Fig. 3A, see Materials and Methods). This approach allowed us to compare abundance of THRAP3–RNA complexes relative to size-matched protein-bound RNA input within each genotype, thus controlling for alterations in total protein-bound RNA abundance that may occur between genotypes. We found that THRAP3 binding in the *Bmal1*^−/−^ cells was enriched relative to *Bmal1*^−/−^ size-matched input specifically within coding exons flanking exons that were skipped more frequently in *Bmal1*^−/−^ cells (Fig. 3A), but not in regions surrounding exons that were preferentially retained in clock mutant cells (Supplemental Fig. S6B), as indicated by increased normalized sequencing reads in THRAP3 IP versus size-matched input control at the exons immediately up-stream of and down-stream from the skipped exon (Fig. 3A). In contrast, we did not observe enrichment of THRAP3 IP sequencing reads relative to input in WT cells in these regions (Fig. 3A), suggesting that THRAP3 displays increased occupancy along these regions in *Bmal1*^−/−^ cells compared with WT. This pattern of enrichment of THRAP3 IP reads compared with input can also be visualized within individual genes containing clock-dependent SE events, including *Cask*, *Madd,* and *Dctn1* (Fig. 3B; Supplemental Fig. S6C). Given that THRAP3 displays increased binding at exons flanking differentially spliced cassette exons in *Bmal1*^−/−^ β cells, we hypothesized that siRNA-mediated depletion of *Thrap3* in *Bmal1*^−/−^ β cells would lead to increased inclusion of the skipped exons. Indeed, we observed increased retention of skipped cassette exons in *Madd*, *Cask*, and *Dock11* by ∼1.5-fold in *Bmal1*^−/−^ cells expressing *Thrap3* siRNA (Fig. 3C; Supplemental Fig. S6D), without altering cassette exon inclusion in WT cells (Supplemental Fig. S6E). However, THRAP3 depletion did not restore inclusion of the cassette skipped in *Dctn1*, which may be regulated by other clock-controlled RBPs (Supplemental Fig. S5G). This data indicates that increased THRAP3 occupancy at these RNAs in *Bmal1*^−/−^ cells drives exon skipping.

Having established a role for THRAP3 in AS in *Bmal1*^−/−^ cells, we next hypothesized that THRAP3 may contribute to the rhythmic AS in WT islets. To test whether THRAP3 was enriched within exons flanking rhythmic cassette exons in a time of day-dependent manner, we examined SE events displaying significant changes between the two time points containing the largest number of rhythmic splicing events (44 and 56 h after shock). Similar to regions containing SEs identified in *Bmal1*^−/−^ cells, exons frequently skipped at 44 h after shock displayed THRAP3 enrichment relative to input RNA in *Bmal1*^−/−^ cells at the exons flanking the skipped exon cassette (Supplemental Fig. S6F), indicating that the molecular clock regulates its deposition on RNA in proximity to clock-controlled cassette exons.

In addition to its role as an RNA-binding protein, THRAP3 can also be recruited to chromatin by CLOCK/BMAL1 and PPARγ (Lande-Diner et al. 2013; Choi et al. 2014). To determine whether loss of BMAL1 impacts THRAP3 chromatin binding in β cells, we performed THRAP3 chromatin immunoprecipitation sequencing (ChIP-seq). We found a greater number of THRAP3 ChIP-seq peaks in *Bmal1*^−/−^ cells (12,988) compared with WT cells (6167); however, THRAP3 occupancy at peaks identified in each genotype was tightly correlated between genotypes (Supplemental Fig. S7A). Next, since THRAP3 ChIP-seq peaks occurred in 57% (533/942) of genes that were alternatively spliced in *Bmal1*^−/−^ cells, and since THRAP3 is corecruited to target genes by collaborative TFs (Lande-Diner et al. 2013; Choi et al. 2014), we compared TF motifs that were enriched within the promoters of the following gene sets: (1) genes with THRAP3 ChIP-seq peaks in their promoters, (2) genes alternatively spliced in *Bmal1*^−/−^ β cells, and (3) genes with rhythmic AS events in synchronized islets. Motif analysis within THRAP3-bound promoters of rhythmically spliced genes revealed enrichment of circadian E-box, bZIP, and ETS motifs (Fig. 3D; Fang et al. 2014). Notably, mRNAs encoding several TFs predicted by Homer to bind these motifs, including E-box-binding bHLH proteins *Usf2, Tfe3*, and *Maz*, bZIP protein c*Jun*, and ETS family proteins *Elk1* and *Elf1*, were up-regulated in clock mutant β cells, suggesting that they may be responsible for clock-dependent THRAP3 recruitment to target genes (Supplemental Fig. S7B). Moreover, THRAP3 binding density, computed by plotting normalized ChIP-seq reads along indicated genomic regions, was enriched in the vicinity of previously published CLOCK and BMAL1 ChIP-seq peaks occurring within promoters of β-cell genes (Supplemental Fig. S7C). However, THRAP3 was not enriched in binding within promoter ChIP-seq peaks for the noncircadian β-cell lineage determining factor PDX1 (Supplemental Fig. S7C; Perelis et al. 2015). Together these data suggest that recruitment of THRAP3 by the core molecular clock and coregulated TFs is coupled to alternative splicing.

### Alternative splicing of *Cask* and *Madd* directly modulates β-cell insulin secretion

**Figure 4.**
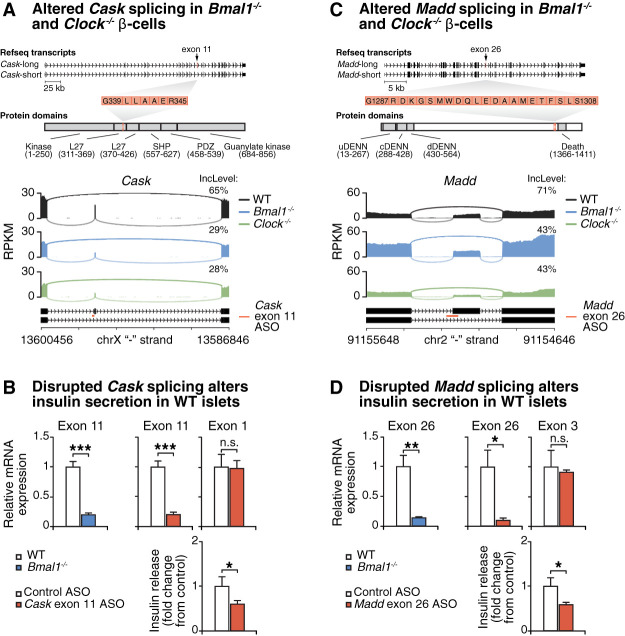
Skipped exons within *Cask* and *Madd* in circadian mutant β cells impair insulin secretion. (*A*) Refseq transcripts depict the “long” and “short” (i.e., skipping exon 11) isoforms of *Cask*. Exon 11 encodes amino acids 339–345, which InterPro annotates as part of the C-terminal L27 domain. Sashimi plots of the differentially spliced *Cask* gene in *Bmal1*^−/−^ and *Clock*^−/−^ β-cell lines. (*B*) Expression of *Cask* skipped exon 11 or nontargeted exon 1 in WT (*n* = 7) and *Bmal1*^−/−^ (*n* = 6) β-TC6 cells and following control (*n* = 5) and targeting (*n* = 3–5) ASO treatment in WT islets assessed via qPCR. Insulin secretion in the targeted islets, *Cask* ASO-treated (*n* = 17 mice), compared with control ASO-treated (*n* = 11 mice). (*C*) RefSeq transcripts depict the “long” and “short” (i.e., skipping exon 26) isoforms of *Madd*. Exon 26 encodes amino acids 1287–1308, which InterPro annotates as part of a disordered region 58 amino acids upstream of its C-terminal death domain. Sashimi plots of the differentially spliced *Madd* gene in *Bmal1*^−/−^ and *Clock*^−/−^ β-cell lines. (*D*) Expression of *Madd* skipped exon 26 or nontargeted exon 3 in WT (*n* = 7) and *Bmal1*^−/−^ (*n* = 6) β-TC6 cells and following control (*n* = 5) and targeting (*n* = 4) ASO treatment in WT islets assessed via qPCR. Insulin secretion in the targeted islets, *Madd* ASO-treated (*n* = 15 mice) compared with control ASO-treated (*n* = 9 mice).

Given that *Cask* and *Madd* display among the highest degrees of exon inclusion level difference in *Bmal1*^−/−^ and *Clock*^−/−^ cells compared with WT (Fig. 2C,D), and that THRAP3 is enriched at exons flanking the skipped exon in *Cask* and *Madd* in *Bmal1*^−/−^ cells (Fig. 3B), we sought to determine whether missplicing of *Cask* and *Madd* contributes to impaired β-cell function in *Bmal1*^−/−^ cells. *Cask* had an ∼36% reduction in inclusion of exon 11 in both *Bmal1*^−/−^ and *Clock*^−/−^ cells (Fig. 4A), and *Madd* displayed a 28% reduction in exon 26 in both *Bmal1*^−/−^ and *Clock*^−/−^ cells (Fig. 4C). CASK was of particular interest as a member of the membrane-associated guanylate kinase (MAGUK) protein family that helps initiate assembly of the presynaptic secretory machinery. CASK is involved in exocytosis of neurotransmitters in the brain (Atasoy et al. 2007) and of insulin in the endocrine pancreas (Suckow et al. 2008; Wang et al. 2015). Furthermore, Uniprot and Interpro annotations show that exon 11 of *Cask*, which is skipped more frequently in the circadian mutants, encodes a domain that facilitates interactions with synaptic membrane proteins (Fig. 4A; Chetkovich et al. 2002; Lee et al. 2002; Roh et al. 2002). *Madd*, a splice variant of the insulinoma-glucagonoma clone 20 (*IG20*) gene, contains a single nucleotide polymorphism associated with human type 2 diabetes, and *Madd* knockout mice display hyperglycemia and impaired insulin secretion (Li et al. 2014). Exon 26 of *Madd*, which is skipped more often in the clock mutants, encodes 58 amino acids upstream of its death domain, which inhibits caspase activation (Fig. 4C; Kurada et al. 2009). To examine the impact of the increased abundance of the short isoforms of *Cask* and *Madd* transcripts in *Bmal1* and *Clock* mutant β cells, we generated antisense oligonucleotides (ASOs) targeting the exons that were skipped more frequently in the mutants. ASOs are short single-stranded segments of DNA that can affect expression of alternatively spliced protein isoforms by initiating exon skipping or inclusion by masking silencing sites during splicing. We treated WT islets with control and targeting ASOs against *Cask* and *Madd* and observed ∼81% and ∼90% reduction in inclusion of *Cask* exon 11 and *Madd* exon 26 mRNA, respectively, relative to a nontargeting control ASO (Fig. 4B,D), while the ASOs did not alter expression of upstream exons 1 and 3 in *Cask* and *Madd,* respectively (Fig. 4B,D). Notably, islets treated with targeting ASOs against both *Cask* and *Madd* displayed significantly reduced glucose-stimulated insulin secretion compared with control ASO-treated islets (by ∼40%) (Fig. 4B,D), suggesting that clock-regulated splicing of these transcripts supports β-cell function. Thus, these results demonstrate that mimicking exon skipping through the use of ASOs, which promotes expression of the short isoforms of these two genes whose splicing is controlled by the circadian clock, reduces glucose-stimulated insulin secretion, suggesting that missplicing of these mRNAs contributes to secretory failure in the setting of circadian disruption.

### Alterations in both transcription and alternative splicing of *Snap25a* in circadian mutant and WT β cells

Analysis of RNA-seq by rMATS of circadian mutant cells revealed that *Snap25* was one of the few genes that was both differentially expressed (74% and 54% reduction in mRNA in *Bmal1*^−/−^ and *Clock*^−/−^ cells, respectively, compared with controls) and spliced (MXE: 15% and 25% reduced inclusion of alternative exon 5b versus 5a in *Bmal1*^−/−^ and *Clock*^−/−^ cells) (Fig. 2A,C,D; Supplemental Figs. S5D, S8A). Since SNAP25 is a key component of the core SNARE complex that mediates stimulus-dependent release of insulin from pancreatic β cells and overall expression levels were reduced in circadian mutant β cells, we first tested whether overexpression of either of the two major splice-variant isoforms of SNAP25 could restore secretion in clock mutant β cells. We generated stable *Bmal1*^−/−^ and WT β-TC6 cell lines overexpressing full-length human *Snap25a* or *Snap25b* cDNAs, which were validated by qPCR and Western blot (Supplemental Fig. S8B). Surprisingly, overexpression of the *Snap25a* isoform, which was more abundant relative to *Snap25b* in clock mutant cells, restored glucose-stimulated insulin secretion in *Bmal1*^−/−^ cells to levels similar to the ones observed in WT controls (Supplemental Fig. S8B). However, overexpression of the *Snap25b* did not rescue insulin secretion in the *Bmal1*^−/−^ cells despite high levels of overexpression (Supplemental Fig. S8B). Overexpression of either *Snap25a* or *Snap25b* above endogenous levels in WT cells did not alter insulin secretion, indicating that reduced transcription of *Snap25*, as opposed to reduced AS-mediated selection of the *Snap25b* isoform in *Bmal1*^−/−^ β cells, contributes to impaired insulin secretion. To next test whether loss of either the *Snap25a* or *Snap25b* isoforms per se was associated with altered GSIS independently from differences in underlying transcription, we used ASOs to block inclusion of alternative exon 5a or 5b and observed 90% and 83% reduction in exons 5a and 5b, respectively (Supplemental Fig. S8C). Importantly, ASO treatment did not alter the total transcript abundance of either isoform. We observed that loss of the *Snap25a* transcript resulted in a 78% reduction in insulin secretion in WT islets, while loss of the *Snap25b* isoform did not decrease the insulin response (Supplemental Fig. S8C). Thus, our studies suggest that reduced overall *Snap25* expression levels, as opposed to reduced inclusion of alternative exon 5b, contribute to impaired insulin secretion in clock mutant β cells.

### Diet-induced obesity and circadian disruption impair shared exocytic pathways that are controlled by AS

Having established that rhythmic AS impacts key regulators of exocytosis in circadian mutant conditions, we sought to determine whether impaired AS might represent a more general defect associated with β-cell failure in the setting of type 2 diabetes. To determine whether impaired alternative pre-mRNA splicing might occur under conditions of β-cell disruption in type 2 diabetes and/or obesity, we analyzed existing deep RNA sequencing data from islets isolated from high-fat diet (HFD)-fed compared with regular chow-fed mice (CON; >100 million reads per sample; GSE92602) (Motterle et al. 2017). Principle component analysis (PCA) clustering revealed that AS events in islets from HFD-fed mice segregate from control-fed mice (Supplemental Fig. S9A). Furthermore, we detected 259 differential AS slice events, including 170 SEs, 30 MXEs, 27 A5SS, and 32 A3SS, among 228 unique genes (Δψ > 0.10, *P* < 0.05) (Supplemental Fig. S9B; Supplemental Table S5), while there were 187 genes differentially expressed (log_2_ FC > 1, FDR < 0.05). Interestingly, similar to the clock mutant islets, there was little overlap between differentially spliced and differentially expressed genes in the islets from HFD-fed mice (2.6%) (log_2_ FC > 1, FDR < 0.05). Of particular interest, the ortholog of the recently identified type II diabetes risk gene *PAM* (Thomsen et al. 2018) in humans was one of the most differentially spliced transcripts in the islets from HFD-fed mice (Supplemental Fig. S9B) despite similar total *Pam* expression in these data. This further provides evidence toward splicing as an inherent regulatory mechanism through which β-cell function is mediated. To elucidate how these diet-induced changes in RNA processing intersect with circadian control, we next examined the overlap between AS events significantly altered in islets following HFD compared with those altered in the *Bmal1*^−/−^ cell lines (Δψ > 0.10, *P* < 0.05). Surprisingly, we found a 15.8% overlap (36 out of 228 AS HFD genes) between the two groups, including in the *TATA-binding protein* (*Tbp*), the *guanine nucleotide-binding protein* (*G protein*; *Gnas*), and *myosin5* (*Myo5*), and pathway analyses revealed that the most enriched pathways at the intersection of HFD-induced and circadian-regulated splicing act to control membrane protein trafficking and vesicle transport (Supplemental Fig. S9C,D). Overall, the differential AS events were over-represented in gene ontology terms related to synaptic vesicle transport and peptide secretion (Supplemental Fig. S5B).

## Discussion

Mounting evidence from genomic analyses has established extensive circadian control in the expression of tissue-specific gene networks central to metabolism (Koike et al. 2012; Remsberg et al. 2017; Yeung et al. 2018). Most studies have focused on using mRNA- and nascent RNA sequencing in tandem with assays for measuring TF binding to understand the role of the molecular clock in initiating RNA synthesis (Fang et al. 2014; Zhang et al. 2014). More recent ribosome-associated RNA sequencing and proteomics have demonstrated that rhythmicity and phase in circadian transcription does not necessarily reflect final protein expression, particularly outside of the core circadian activator and repressor proteins (Deery et al. 2009; Jang et al. 2015; Janich et al. 2015). Emergence of clock function has been implicated in β-cell maturation (Alvarez-Dominguez et al. 2020), and circadian control of splicing events are necessary for the proper assembly of synaptic machinery in developing neurons (Song et al. 2017; Wang et al. 2018). Despite this link between the circadian clock and neuronal cell-specific splicing, whether the circadian clock mediates AS across the 24-h cycle in peripheral tissues remains incompletely understood.

Here we identified pre-mRNA alternative splicing as an output of the molecular clock that contributes to insulin secretion in pancreatic β cells. We also showed that disrupted AS of key exocytic genes following circadian disruption contributes to impaired β-cell function. Our analyses of RNA sequencing coverage of splice junctions demonstrated extensive modulation of the circadian transcriptome in islets at the level of mRNA isoform selection, and that genes encoding proteins mediating exocytosis, synaptic transmission, and transcription are rhythmically alternatively spliced (Fig. 1; Supplemental Fig. S1). Global splicing patterns further indicated that a majority of splicing events involve retention or exclusion of individual cassette exons (SE), and that these splicing events display a bimodal distribution across the circadian cycle, peaking at times corresponding roughly to the zenith and nadir of endogenous clock-driven insulin secretion (Fig. 1; Supplemental Fig. S1). Our observations suggest that the molecular clock within pancreatic β cells regulates insulin secretion by ensuring appropriate mRNA isoform selection at specific phases throughout the day/night cycle.

Since the functional consequences of clock gene manipulation to date have largely been performed in primary murine and human pancreatic islets containing mixed endocrine cell types, our analyses of clonal *Bmal1* and *Clock* knockout β-cell lines enabled the interrogation of clock-dependent transcriptional and AS events associated with impaired insulin secretion in pure populations of insulin-secreting cells (Fig. 2; Supplemental Fig. S2). Genetic depletion of *Bmal1* and *Clock* resulted in phenotypic changes comparable with those observed in pancreas-specific knockout mouse models, such as refractory secretory responses to both glucose and nonnutrient insulin secretagogues (Fig. 2; Supplemental Figs. S2–S4). Moreover, electrophysiologic analysis of *Bmal1* mutant β-cell lines and mouse β cells revealed a conserved defect in depolarization-evoked exocytosis (Fig. 2B), localizing the secretory defect to the final stages of insulin granule fusion and peptide release.

The transcriptomes of *Bmal1*^−/−^ and *Clock*^−/−^ β cells revealed perturbations in mRNAs involved with peptide secretion and second messenger signals that trigger insulin secretion (Fig. 2A). We detected significant changes in the AS of ∼100 genes, including vesicle membrane components, kinases, GTPases responsible for posttranslational modifications to the secretory apparatus, and plasma membrane proteins that are also important regulators of neuronal maturation and synaptic function (Fig. 2D). A majority of these missplicing events occurred in mRNAs that were not differentially expressed in the circadian mutants, highlighting the importance of clock control of isoform selection throughout the day. Our analyses of oscillating RNA transcripts in synchronized WT islets, synchronized WT and clock deficient β cells, and islets from hyperglycemic animals reveal over-representation of genes encoding vesicle trafficking and synaptic proteins. Furthermore, rhythmic- and CLOCK/BMAL1-dependent transcriptomes revealed a repertoire of dynamic RBPs that mediate AS. There is evidence for RBPs regulating circadian splicing events (Benegiamo et al. 2018; Wang et al. 2018) and insulin secretion (Juan-Mateu et al. 2017), but little is known about how circadian RBPs and downstream rhythmic RNA processing regulates peripheral tissue function. Here we demonstrated that the circadian RBP THRAP3 regulates cassette exon skipping in β cells at transcripts controlling insulin secretion, including *Cask* (a scaffold for calcium-calmodulin kinase) and *Madd* (a RAB3 GTPase-activating protein). We show that proper *Thrap3* expression is necessary for maintaining exon skipping in *Cask* and *Madd* in *Bmal1*^−/−^ cells. Surprisingly, simply mimicking the SE events found in *Bmal1*^−/−^ cells in *Cask* and *Madd* transcripts was sufficient to suppress glucose-stimulated insulin secretion in WT mouse islets, suggesting that missplicing of mRNAs such as *Cask* and *Madd* may contribute to secretory failure with circadian disruption (Fig. 4). It is intriguing to speculate that reduced inclusion of the *Cask* exon 11 in the circadian mutants leads to altered interactions with key synaptic membrane proteins such as Postsynaptic density protein 95 (PSD-95), Neurexin-1, and Munc-18-interacting protein (MINT) (Chetkovich et al. 2002; Mukherjee et al. 2008), in turn impeding vesicle trafficking (Gillespie and Hodge 2013). Further analyses will be required to determine the impact of reduced inclusion of these *Cask* and *Madd* exons on vesicle trafficking and calcium signaling within circadian mutant β cells. These findings reveal the importance of circadian control of AS of key exocytotic-controlling genes in the maintenance of β-cell function.

*Snap25* was one of only four genes that were both differentially spliced and reduced in expression in both *Clock*^−/−^ and *Bmal1*^−/−^ β cells. *Snap25* encodes a target membrane SNARE protein critical for synaptic vesicle exocytosis and expresses either of the mutually exclusive exons 5a or 5b, which arose as a consequence of an ancestral duplication event (Bark 1993). While both SNAP25a and SNAP25b are capable of assembling functional SNARE complexes in pancreatic β cells (Daraio et al. 2017), the relative roles of the *Snap25a* and *Snap25b* isoforms are not well understood. We observed reduced expression of both the *Snap25a* and *Snap25b* isoforms in the circadian mutant β cells, with a greater reduction in the *Snap25b* isoform relative to the *Snap25a* isoform (Supplemental Fig. S8). Our findings that *Snap25a* overexpression enhanced insulin secretion in the circadian mutant β cells, and that blocking inclusion of the *Snap25a* but not *Snap25b* isoform using ASOs inhibited insulin secretion, together suggests the β-cell clock modulates both splice isoform selection and transcription of rhythmic genes. Our studies verify the observation that *Snap25a*, which is a less functional fetal isoform in neurons, is coexpressed with *Snap25b* in β cells and enhances insulin secretion (Gonelle-Gispert et al. 1999; Daraio et al. 2017). Finally, observations that (1) AS of *Snap25* and other neuronal mRNAs is developmentally regulated (Bark et al. 1995; Johansson et al. 2008; Song et al. 2017) and (2) the emergence of circadian rhythms during human β-cell maturation coincides with metabolic maturation (Alvarez-Dominguez et al. 2020), raise the possibility that the circadian control of AS may participate in β-cell maturation.

It is estimated that AS occurs in ∼94% of human genes (Wang et al. 2008) and that up to 30% of AS occurs in a tissue-specific manner (Pan et al. 2008). We found that circadian disruption and high-fat feeding led to disrupted AS of an overlapping set of vesicle trafficking and exocytic RNA networks in islets (Supplemental Fig. S9), suggesting that AS is perturbed in both circadian- and diet-induced metabolic disease. However, the RNA-binding proteins (RBPs) regulating AS during circadian and metabolic stress have not been identified (Supplemental Tables S1, S3, S4). Here we identified THRAP3 as a key splicing-regulatory RBP important in rhythmic β-cell physiology. While THRAP3 depletion in clock-deficient cells partially restores cassette exon inclusion, we note that *Thrap3* siRNA treated *Bmal1*^−/−^ cells still displayed reduced cassette exon inclusion levels relative to WT cells and *Thrap3* knockdown did not affect all differentially spliced transcripts, such as *Dctn1*. Therefore, it is likely that a synergistic relationship exists between clock-controlled and rhythmic RBPs (Supplemental Fig. S5G). We observed that α and β cells exhibit cell type-specific patterns in AS across the 24-h timescale (Fig. 2E). Therefore, it remains a possibility that cell type-specific phases of TF recruitment and rates of transcription may be key to understanding how RBPs are targeted on a circadian timescale. The role of transcriptional regulators in RBP recruitment (Supplemental Tables S1, S3, S4) and the mechanisms by which AS is directed by RBPs in pancreatic β cells remains incompletely understood. Antisense oligonucleotide therapies currently exist for neuromuscular diseases caused by missplicing, such as spinal muscular atrophy (Hua et al. 2011; Passini et al. 2011), and new chemistries now enable the delivery of ASOs specifically to the β cell (Ämmälä et al. 2018). A greater understanding of the molecular mechanisms underlying abnormally spliced transcripts will shed light on therapies for disorders of circadian disruption, including type 2 diabetes.

## Materials and methods

### Animals

Male WT C57BL/6J mice were purchased from the Jackson Laboratory. *PdxCre;Bmal1^flx/flx^* mice (Marcheva et al. 2010) were produced and maintained on a C57BL/6J background at the Northwestern University Center for Comparative Medicine. *Clock^flx/flx^* mice were crossed with *PdxCre* transgenic mice (kindly provided by Dr. David R. Weaver, University of Massachusetts Medical School, and Dr. Douglas Melton, Harvard University, respectively) to generate *PdxCre;Clock^flx/flx^* mice, as well as *Clock^flx/flx^* and *PdxCre* littermate controls (Gu et al. 2002; Debruyne et al. 2006). Offspring from this cross were on a mixed B6x129xICR background. Unless otherwise stated, mice were maintained on a 12:12 light/:dark (LD) cycle and allowed free access to regular chow and water. All animal care and use procedures were conducted in accordance with regulations of the Institutional Animal Care and Use Committee at Northwestern University.

### β-TC6 cell culture

β-TC6 cells were purchased from ATCC (CRL-11506) and cultured in Dulbecco's modified Eagle's medium (DMEM; Gibco) supplemented with 15% FBS (Atlanta Biologicals), 1% L-glutamine (Gibco), and 1% penicillin/streptomycin (Gibco). All cells used in experiments were at <15 passages. For synchronization and knockdown experiments WT and *Bmal1*^−/−^ cells were plated at 2.5 × 10^5^ cells per well on 24-well plates. For siRNA treatment, 10 pmol of nontargeting control siRNA (Dharmacon D-001810-10-05) or Thrap3 Silencer Select siRNA (Thermo Fisher Scientific 4390771) in Lipofectamine (1.5 μL/well), and Opti-MEM medium (300 μL/well) were first added to 24-well plates followed by addition of 2.5 × 10^5^ cells in 1 mL of normal medium. RNA was collected 72 h later with TRI reagent. For synchronization cells were plated given a 10 μM forskolin shock for 1 h, followed by warm PBS wash and medium change to 5% FBS supplemented DMEM. Cells were collected between 24 and 44 h later with TRI reagent.

### CRISPR-mediated *Bmal1* and *Clock* deletion in β-TC6 cell culture

Exon 8 of *Bmal1* and exons 6 and 7 of *Clock* were deleted in β-TC6 cells by CRISPR–Cas9 and homology-directed repair (HDR). For *Bmal1*, intronic DNA flanking *Bmal1* exon 8 was cloned into pTOPO2.1 (pBmal1-HR) (Invitrogen), and cells were cotransfected with guide RNA, Cas9 [pSpCas9(BB)-2A-Puro; Addgene], and pBmal1-HR plasmids. Stably integrated clones were selected for neomycin resistance (G418, Mediatech), and single colonies were hand picked and cultured individually. RNA and protein were extracted from these colonies and *Bmal1* mRNA and protein were assessed by qPCR and Western blot. For *Clock*, cells were cotransfected with guide RNA, Cas9 (Clock CRISPR/Cas9 KO plasmids; Santa Cruz Biotechnology), and Clock HDR plasmids (Santa Cruz Biotechnology sc-419693-HDR). Stably integrated clones were selected for puromycin resistance (puromycin dihydrochloride, Sigma-Aldrich), and single colonies were hand picked and cultured individually. RNA and protein were extracted from these colonies and *Clock* mRNA and protein were assessed by qPCR and Western blot.

### Bioluminescence monitoring in *Bmal1*^−/−^ and *Clock*^−/−^ β-cell lines

β-TC6 WT, *Bmal1*^−/−^, and *Clock*^−/−^ cells were infected with a lentivirus expressing a *Per2-dLuc* reporter construct (gift from A. Liu, University of Memphis) (Liu et al. 2008) and maintained in DMEM with 10% FBS and 2.5 µg/mL blastocidin (Sigma) to select for stable *Per2-dLuc* integration. Sealed cell cultures were incubated in 1.2 mL of DMEM containing 352.5 µg/mL sodium bicarbonate (Gibco), 10 mM HEPES (Gibco), 2 mM L-glutamine, 1% FBS, 1% penicillin/streptomycin, 1% sodium pyruvate (Corning), and 0.1 mM luciferin sodium salt (Biosynth AG). Cultures were placed in a LumiCycle luminometer (Actimetrics), temperature was synchronized for 24 h (sine wave: 37°C–41°C–37°C–33°C–37°C), and plates were then maintained at 37°C. Bioluminescence was recorded continuously for several days.

### Glucose and insulin measurements

Blood glucose and plasma insulin levels in *ad libitum*-fed mice were assessed at ZT2 and ZT14 from tail vein bleeds. Glucose tolerance tests were performed in mice that were first fasted for 14 h. Blood glucose and insulin levels were measured at the indicated times following intraperitoneal (IP) injection of glucose (2 or 3 g/kg body weight, as indicated). Plasma insulin levels were measured by ELISA (Crystal Chem, Inc.).

### Pancreatic islet and cell line insulin secretion assays

Pancreatic islets were isolated via bile duct collagenase digestion (*Collagenase P*, Sigma) and Biocoll (Millipore) gradient separation and left to recover overnight at 37°C in RPMI 1640 with 10% FBS, 1% L-glutamine, and 1% penicillin/streptomycin. For insulin release assays, five equally sized islets per mouse (in duplicate) were statically incubated in Krebs-Ringer buffer the following morning (∼ZT6) and stimulated for 1 h at 37°C with 2 or 20 mM glucose or 30 mM KCl. Supernatant was collected and assayed for insulin content by ELISA. Islets were then sonicated in acid-ethanol solution and solubilized overnight at 4°C before assaying total insulin content by ELISA. Percent insulin content secreted as a response to stimulus was normalized to basal secretion levels for each condition and reported as fold change from control. For insulin release assays in cell lines, 3-4 × 10^6^ cells were cultured for 3 d in 6-cm suspension culture dishes. Formed pseudoislets were then transferred into new suspension culture media and left to recover overnight. Glucose-responsive insulin secretion was performed as described above, using 10 pseudoislets per sample and a basal glucose level of 0 mM glucose instead of 2 mM.

### Immunohistochemistry

Mice were anesthetized with IP injection of phenobarbital (50 mg/mL; Nembutal) and perfused with heparinized saline, followed by 4% paraformaldehyde (Sigma) in PBS. Pancreata were removed, postfixed with 4% PFA overnight at 4°C embedded in paraffin, and blocks of 6-μm sections were mounted on slides. The following primary antibodies were used for staining: guinea pig anti-insulin (1:500; DAKO), mouse antiglucagon (1:500; Sigma), and rabbit anti-CLOCK (1:500; Millipore). Secondary antibodies included AMCA goat antiguinea pig (1:400; Jackson ImmunoResearch), Alexa fluor 488-conjugated goat antimouse (1:400, Invitrogen), and Alexa flour 546-conjugated goat antirabbit (1:400; Life Technologies). Images were acquired with PictureFrame 1.0 using a Zeiss Axioskop 50.

### MRI measurements

Body composition of live mice was determined by quantitative NMR. Data were analyzed using software by EchoMRI technology (Echo Medical Systems). Fat and lean mass was reported as percentage of body weight.

### Activity and feeding measurements

Locomotor activity was monitored in 2- to 4-mo-old pancreas-specific *Clock* knockout mice and their respective littermate controls. All animals were individually housed in standard mouse cages equipped with infrared sensors and allowed free access to food and water. Mice were placed in a 12:12 LD cycle for 15 d, followed by 15 d in constant darkness (DD). Total activity counts were quantified as the total number of infrared sensor beam breaks (Chronobiology kit, Stanford Software Systems). Activity data were analyzed in 6-min bouts using ClockLab software (Actimetrics). The free-running period was determined as the duration of time between the major activity periods on consecutive days. Period was calculated using a χ^2^ periodogram for days 20–30 (Sokolove and Bushell 1978). Daytime and nighttime food consumption of individually housed animals with free access to water and regular chow was determined by manual measurement of remaining diet at both ZT0 and ZT12 for five consecutive days.

### RNA isolation for qPCR mRNA quantification

Islets or cells were treated with TRI reagent (Sigma Aldrich) and frozen at −80°C. RNA was isolated according to the manufacturer's protocol and purified using RNeasy columns (Qiagen). cDNAs were then synthesized using the high-capacity cDNA reverse transcription kit (Applied Biosystems). Real-time quantitative polymerase chain reaction (qPCR) analysis was performed using SYBR Green master mix (Applied Biosystems) and analyzed using an Applied Biosystems 7900 Fast real-time PCR system. Relative expression levels were determined using the comparative CT method to normalize target gene mRNA to *β-actin*. Exon-specific primer sequences for qPCR are listed in Supplemental Table S6.

### RNA isolation, library preparation, and RNA sequencing

CRISPR-edited and control cell lines (1 × 10^6^ to 2 × 10^6^) were harvested from six-well plates in triplicate in TRI reagent (Sigma Aldrich), and RNA was isolated using Direct-zol miniprep columns (Zymo) according to the manufacturer's specifications. mRNA libraries for Illumina sequencing were prepared using poly(A) mRNA magnetic isolation module and NEBNext Ultra II directional RNA library kits (New England Biolabs) from 250 ng of total RNA. Libraries were sequenced on an Illumina Nextseq 500 instrument to a minimum of 2 × 10^7^ paired-end 75-bp reads. Raw sequencing reads were mapped to the mm10 genome using annotations from the gencode vM10 assembly with STAR version 2.5.0 (Dobin et al. 2013) using default parameters. Individual transcripts annotated in the gencode vM10 assembly were quantified using featureCounts version 1.5.3 (Liao et al. 2014) and the option “–t exon” was specified to count mRNA features.

### Differential expression and splicing analyses

For differential mRNA expression, DESeq2 (version 1.22.1) (Love et al. 2014) analysis was performed on control, *Bmal1*, and *Clock* knockout cell lines specifying a false discovery cutoff of 0.01 for independent filtering. Differentially expressed transcripts were defined as having a minimum of log_2_ fold change value >1 and false discovery rate-adjusted *P*-value < 0.01. For alternative splicing analyses, rMATS (version 4.0.2) (Shen et al. 2014) was used to identify AS events by quantifying exon–exon junction spanning reads on annotated splice junctions present in the mouse gencode vM10 assembly. Differentially spliced mRNAs were defined as having a false discovery rate of <0.05 and a minimum inclusion level difference >10%.

### Antisense oligonucleotide experiments

Phosphorodiamidate morpholino antisense oligonucleotides (ASOs) were designed according to described guidelines (GeneTools) and synthesized as 25-mer vivo morpholinos by GeneTools. ASOs were resuspended in ultrapure water to stock concentration of 0.5 mM. Specific sequences and their exon targets are listed in Supplemental Table S6. A nontargeting vivo morpholino ASO standard control was purchased from GeneTools and used at final concentrations matching those of targeting ASOs. WT islets were incubated for 4 h in 5% serum medium with ASOs for *Madd* (5 μM) and *Cask* (2.5 μM), and were then transferred to normal culture medium for 24 h before RNA collection or GSIS experiments. For *Snap25* experiments, islets were incubated for 24 h in 5% serum medium with ASOs for *Snap25a* or *Snap25b* (1 μM) before RNA collection or GSIS.

### *Snap25* exon overexpression

WT and *Bmal1*^−/−^ β-TC6 cells were infected with lentivirus expressing either control vector or the complete ORF of the human *Snap25a* or *Snap25b* gene (OriGene PS100092V5, RC202068L3V, and RC212596L3V). Stably integrated clones were selected for puromycin resistance (Sigma) and assayed for *Snap25a/b* mRNA and SNAP25 total protein levels.

### Western blotting

Whole-cell lysates were isolated by treating cell pellets with RIPA buffer (Sigma) containing 1× protease and 1× phosphatase inhibitors (Roche). Protein levels were quantified using DC protein assay (Bio-Rad). Protein extracts were then subject to SDS-PAGE gel electrophoresis and transferred to nitrocellulose (GE Healthcare) or Immobilin-P transfer membrane (Millipore). Primary antibodies used were β-ACTIN (CST 4970), BMAL1 (Santa Cruz Biotechnology sc-48790), CLOCK (Santa Cruz Biotechnology sc-25361), and SNAP25 (CST D9A12).

### Patch-clamp electrophysiology

Patch-clamp measurement of exocytotic responses in single β-TC6, or mouse β cells, was performed at 32°C–35°C using the standard whole-cell technique with the sine + DC lock-in function of a HEKA EPC10 amplifier and PatchMaster software (HEKA Electronics). β-TC6 cells were preincubated in 0 mM glucose EC solution for 1 h, patch-clamped, stimulated with a train of 10 depolarization steps, and the cumulative capacitance was recorded at 20 mM glucose extracellular (EC) solution. Mouse islets were dispersed by shaking in cell dissociation buffer (Gibco, Thermo Scientific) and plated in 35-mm culture dishes. β Cells were identified by cell size and sodium channel inactivation at −90 mV following the experiment, as previously described (Dai et al. 2012). Islet cells were preincubated at 2.8 mM glucose EC for 1 h and capacitance was measured at 10 mM glucose as described above. The pipette solution for depolarization-stimulated capacitance measurements contained 125 mmol/L Cs-glutamate, 10 mmol/L CsCl, 10 mmol/L NaCl, 1 mmol/L MgCl_2_ 6 mmol/L H_2_O, 0.05 mmol/L EGTA, 5 mmol/L HEPES, 0.1 mmol/L cAMP, and 3 mmol/L Mg ATP (pH 7.15 with CsOH). The extracellular bath contained 118 mmol/L NaCl, 20 mmol/L tetraethylammonium chloride, 5.6 mmol/L KCl, 1.2 mmol/L MgCl_2_ 6 mmol/L H_2_O, 2.6 mmol/L CaCl_2_, 5 mmol/L glucose, and 5 mmol/L HEPES (pH 7.4 with NaOH). Patch pipettes, pulled from borosilicate glass and coated with Sylgard, had a resistance of 3–4 MΩ when filled with pipette solution; liquid junction potentials were corrected as appropriate. Quantification of the average cumulative increase in the capacitance of 500-msec depolarizations from −70 to 0 mV was calculated. Measurements were normalized to initial cell size and expressed as femtofarad per picofarad (fF/pF). Data analysis was performed using GraphPad Prism (v7.0c). Comparison of multiple groups was done by one- or two-way ANOVA followed by Bonferroni or Tukey posttest. Data are expressed as means ± SEM, where *P* < 0.05 is considered significant.

### ChIP sequencing and analysis

Approximately 1 × 10^7^ cells per replicate were collected and processed for ChIP-seq as published previously (Perelis et al. 2015). Briefly, cells were fixed on 15-cm plates in 2 mM disuccinimidyl glutarate in PBS + 1% DMSO for 30 min, followed by formaldehyde (1% in PBS) cross-linking for 10 min, followed by quenching with addition of glycine to a total concentration of 125 mM glycine. Cells pellets were collected and frozen at −80°C. Cross-linked pellets were twice disrupted by five passages through a needle and syringe in buffer (150 mM NaCl, 5 mM EDTA at pH 8, 50 mM Tris-HCl at pH 8, 0.35% NP-40) in the presence of Roche Mini EDTA-free protease inhibitor cocktail and spun at 600 rcf for 5 min. Rough nuclear pellets were sheared in 1 mL of shearing buffer (1% SDS, 2.5 mM EDTA at pH 8, 50 mM Tris-HCl at p H8) in a Diagenode sonicator for 10 high-power cycles 30 sec on/30 sec off at 4°C. An aliquot for input samples was taken after shearing. Sheared chromatin was diluted 1:10 in dilution buffer (0.01% SDS, 1.1% Triton X-100, 167 mM NaCl, 1.2 mM EDTA at pH 8, 1.67 mM Tris-HCl at pH 8) and 10 μg of anti-THRAP3 antibody (NB100-40848) was added and gently rocked overnight at 4°C. Secondary antirabbit Dynabeads beads were preblocked in 0.1% BSA and used to pull down antibody-bound chromatin, followed by chromatin decross-linking and purification by Qiagen MinElute and library preparation with NEBNext Ultra II library preparation kit for IP and input. Following selection of Sage Pippin PrepHT for adapter-ligated fragments between 200- and 600-bp libraries were amplified for a total of 11 cycles, cleaned using 0.9× Ampure XP bead size selection, quantified by Qubit, analyzed for base pair size and quality by Agilent Bioanalyzer, and pooled for sequencing on an Illumina Nextseq 500 instrument with 75-bp single-end reads with >17 million unique reads aligned per sample. Reads were aligned with Bowtie2 (v2.2.4) to the mm10 genome with default parameters. Peaks were identified for each IP over pooled input for each genotype using HOMER findPeaks command with setting -style factor. Motif enrichment for ChIP peaks and gene lists promoters was performed using the command findMotifGenome.pl and findMotifs.pl, respectively, and percent enrichment scores versus background plotted in R using ggplot2.

### eCLIP sequencing and analysis

eCLIP was performed as described previously (Van Nostrand et al. 2016) with minor modifications. Approximately 4 × 10^7^ cells per replicate were cross-linked to preserve protein–RNA complexes by UV irradiation (254 nm, 400 mJ/cm^2^) and cell pellets were harvested in PBS and snap-frozen. Cross-linked cells were lysed in iCLIP lysis buffer (50 mM Tris-HCl at pH 7.4, 100 mM NaCl, 1% NP-40, 0.1% SDS, 0.5% sodium deoxycholate, 1:200 protease inhibitor cocktail III) supplemented with 440 units of murine RNAse inhibitor (NEB) and subjected to limited RNase 1 digestion (Ambion) for 5 min prior to immunoprecipitation with anti-THRAP3 antibody (NB100-40848). Immune complexes were immobilized on magnetic beads (M-280 sheep antirabbit IgG Dynabeads, Thermo Fisher Scientific) and subjected to stringent washes in high-salt buffer (50 mM Tris-HCl at pH 7.4, 1 M NaCl, 1 mM EDTA, 1% NP-40, 0.1% SDS, 0.5% sodium deoxycholate) and low-salt buffer (20 mM Tris-HCl at pH 7.4, 10 mM MgCl2, 0.2% Tween-20). Following dephosphorylation (FastAP, Thermo Fisher) and T4 PNK treatment (NEB) of protein-bound RNA fragments on magnetic beads, and barcoded RNA adapters were ligated to the 3′ end (T4 RNA Ligase, NEB). Samples were then run on SDS–polyacrylamide protein gels, transferred to nitrocellulose membranes, and a region from 150 to 225 kDa corresponding to THRAP3 and THRAP3–RNA complexes was excised and proteinase K-treated (NEB) to isolate RNA. RNA was reverse-transcribed with SuperScript III (Thermo Fisher) and treated with ExoSAP-IT (Affymetrix) to remove excess oligonucleotides. A second DNA adapter (containing a random mer of five or 10 random bases at the 5′ end) was ligated to the 3′ end of cDNA fragments (T4 RNA Ligase, NEB). After bead cleanup (Dynabeads MyOne Silane, Thermo Fisher), an aliquot of each sample was first subjected to qPCR (to identify the proper number of PCR amplification cycles), and the remainder was PCR-amplified (Q5, NEB) and size-selected via agarose gel electrophoresis. Libraries were sequenced on an Illumina NextSeq500 with 75-bp single-end reads with >15 million uniquely mapped reads aligned to the mouse mm10 reference genome per sample using STAR (v2.4.0i). Metagene plots were produced by first extending relevant AS intron and exon chromosomal locations by 50 bp upstream and downstream, followed by quantification of reads per million mapped reads (RPM) across 100 bins using the Metagene package in Rstudio (v3.6.1).

### Statistical analysis

Where appropriate, results were expressed as mean ± SEM unless otherwise noted. Detailed information on sample size, genotype, and *P*-values can be found within individual figures and figure legends. Statistical analysis was performed by unpaired two-tailed Student's *t*-test unless otherwise indicated. *P* < 0.05 was considered to be statistically significant.

### Sequencing data availability

RNA-seq, ChIP-seq, and eCLIP-seq data sets have been deposited in the Gene Expression Omnibus server accession number GSE146916.

### Competing interest statement

G.W.Y. is cofounder, member of the Board of Directors, on the Scientific Advisory Board, equity holder, and paid consultant for Locanabio and Eclipse BioInnovations. G.W.Y. is a visiting professor at the National University of Singapore. G.W.Y.'s interest(s) have been reviewed and approved by the University of California at San Diego in accordance with its conflict of interest policies. The authors declare no other competing financial interests.

## Supplementary Material

Supplemental Material
