# Supplementary material for: A role for alternative splicing in circadian control of exocytosis and glucose homeostasis
Source: Genes Dev. 2020 Aug 1;34(15-16):1089–105. doi: 10.1101/gad.338178.120 (PMC7397853; doi:10.1101/gad.338178.120)
Supplement: Supplemental Material [file supp_34_15-16_1089__index.html]

A role for alternative splicing in circadian control of exocytosis and glucose homeostasis — Supplemental Material 

# A role for alternative splicing in circadian control of exocytosis and glucose homeostasis

## Supplemental Material

- Supplemental\_Figures.pdf
- Supplemental\_Table\_S1.xlsx
- Supplemental\_Table\_S2.xlsx
- Supplemental\_Table\_S3.xlsx
- Supplemental\_Table\_S5.xlsx
- Supplemental\_Table\_S4.xlsx
- Supplemental\_Table\_S6.xlsx
