# Supplementary material for: A role for alternative splicing in circadian control of exocytosis and glucose homeostasis
Source: Genes Dev. 2020 Aug 1;34(15-16):1089–105. doi: 10.1101/gad.338178.120 (PMC7397853; doi:10.1101/gad.338178.120)
Supplement: Supplemental Material [file supp_gad.338178.120_Supplemental_Figures_.pdf]

Figure S1

A Rhythmic exon inclusion in WT islets

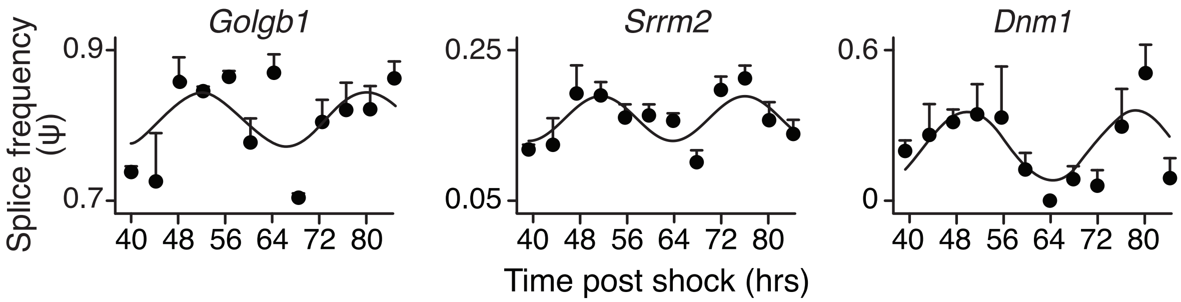

B Unique but related ontology terms enriched among rhythmically spliced vs expressed genes

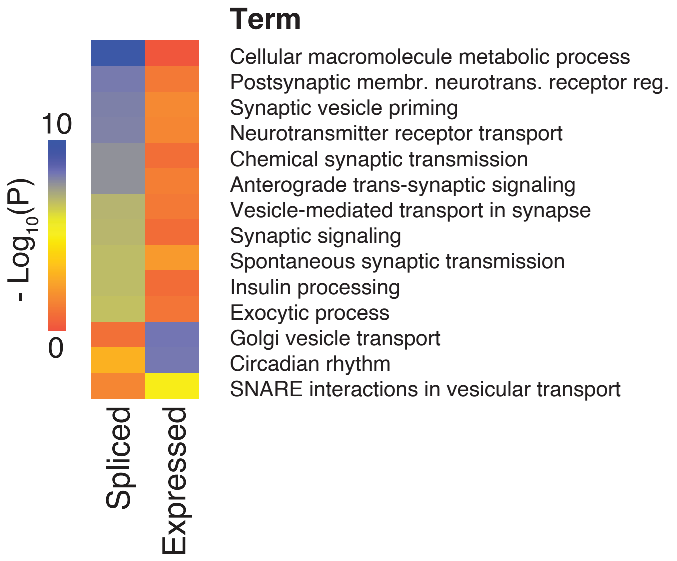

C Peak phases of all cycling RBPs in WT islets

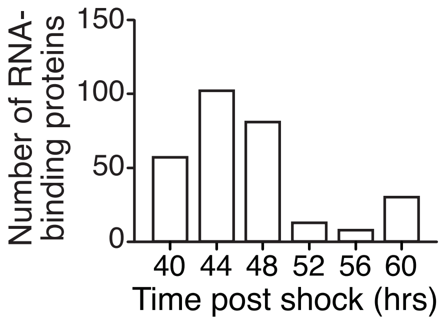

Figure S1. Circadian control of alternative splicing in pancreatic islets throughout the day. (A) Time-of-day-dependent alternative splicing of *Golgb1*, *Srrm2*, and *Dnm1* every 4 hrs across 48 hrs (starting 40 hrs post-forskolin shock) measured by splice frequency (ψ) in oscillating WT islets. **(B)** Pathway analyses reveal significant enrichment of unique but related ontology terms enriched among rhythmically spliced versus rhythmically expressed genes (from (Perelis et al. 2015)). **(C)** Histogram depicting number of rhythmic RBPs with a peak phase at indicated time points.

Figure S2

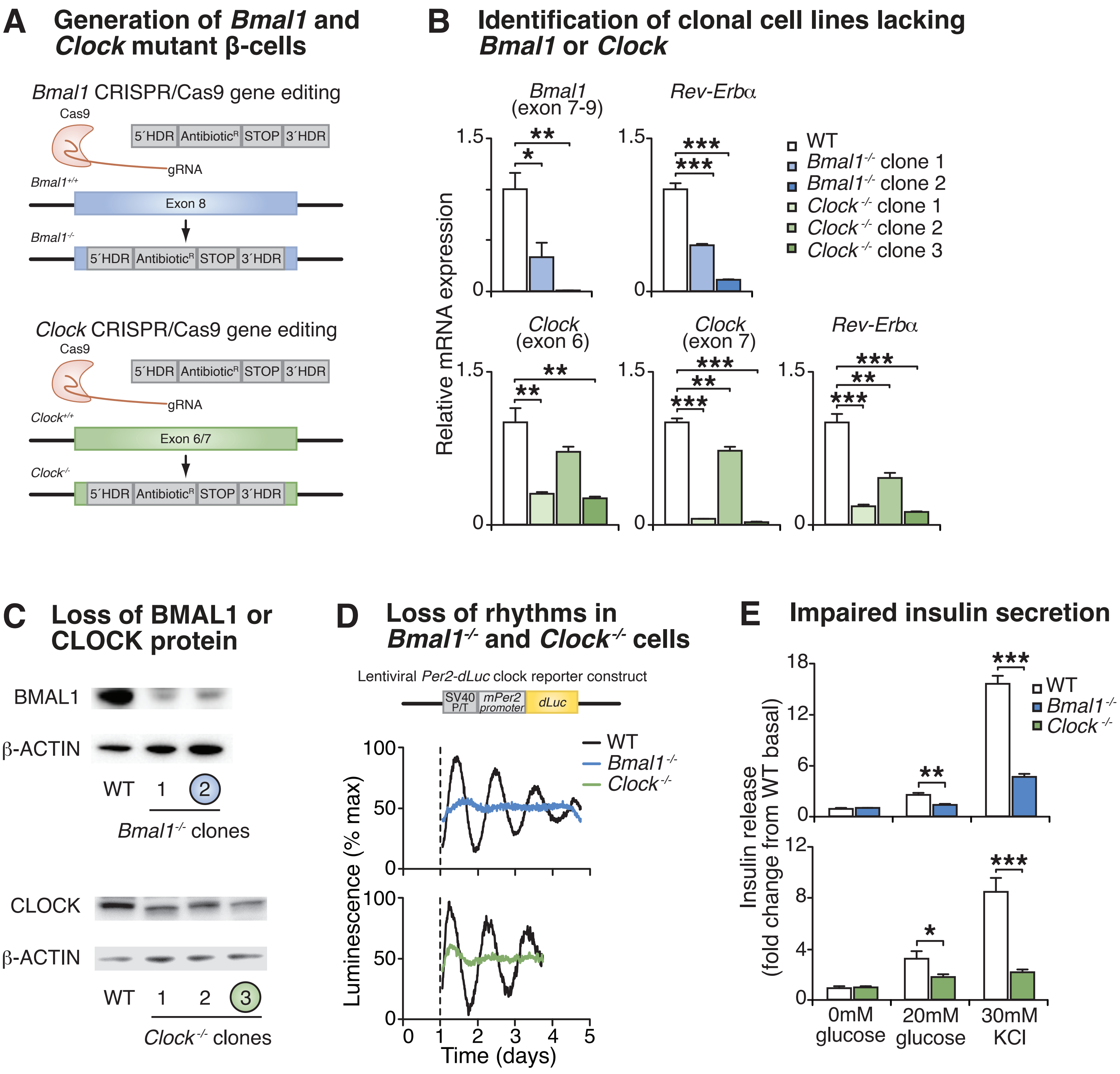
A Generation of Bmal1 and Clock mutant β-cells
Bmal1 CRISPR/Cas9 gene editing
Cas9
5´HDR AntibioticR STOP 3´HDR
gRNA
Bmal1+/+
Exon 8
Bmal1-/-
Clock CRISPR/Cas9 gene editing
Clock+/+
Exon 6/7
Clock-/-
B Identification of clonal cell lines lacking Bmal1 or Clock
Bmal1 (exon 7-9)
Rev-Erbα
Clock (exon 6)
Clock (exon 7)
Relative mRNA expression
1.5
0
WT
Bmal1-/- clone 1
Bmal1-/- clone 2
Clock-/- clone 1
Clock-/- clone 2
Clock-/- clone 3
C Loss of BMAL1 or CLOCK protein
BMAL1
β-ACTIN
WT 1 2
Bmal1-/- clones
CLOCK
WT 1 2 3
Clock-/- clones
D Loss of rhythms in Bmal1-/- and Clock-/- cells
Lentiviral Per2-dLuc clock reporter construct
SV40 P/T
mPer2 promoter
dLuc
WT
Bmal1-/-
Clock-/-
Luminescence (% max)
100
50
0
Time (days)
0 1 2 3 4 5
E Impaired insulin secretion
Insulin release (fold change from WT basal)
18
12
6
0
8
4
0mM glucose
20mM glucose
30mM KCl

Figure S2. Generation of clonal isogenic β-cell lines lacking a functional clock. (A) Schematic of CRISPR/Cas9 gene editing and homology-directed repair (HDR) in mouse Beta-TC6 cells to disrupt the bHLH DNA binding domains of BMAL1 (exon 8) and CLOCK (exons 6/7). **(B)** Quantitative real-time PCR screening of disrupted *Bmal1* and *Clock* exons within individual clones, as well as expression of the downstream clock target *Rev-erbα* (n=3 per genotype). **(C)** BMAL1 and CLOCK protein expression by Western blot within individual *Bmal1*$^{-/-}$ and *Clock*$^{-/-}$ clones. The circle indicates which clones were chosen for use in subsequent experiments. **(D)** Bioluminescence monitoring of PER-LUC expression following synchronization of WT, *Bmal1*$^{-/-}$, and *Clock*$^{-/-}$ β-cell lines expressing a lentivirus encoding the PER2-dLUC reporter. **(E)** Insulin release following exposure to either 20 mM glucose or 30 mM KCl in *Bmal1*- and *Clock*-deficient Beta-TC6 cells compared to controls (n=3 per genotype). Data were analyzed by Student's *t*-test. All values represent mean ± SEM. *p<0.05, **p<0.01, ***p<0.001.

Figure S3

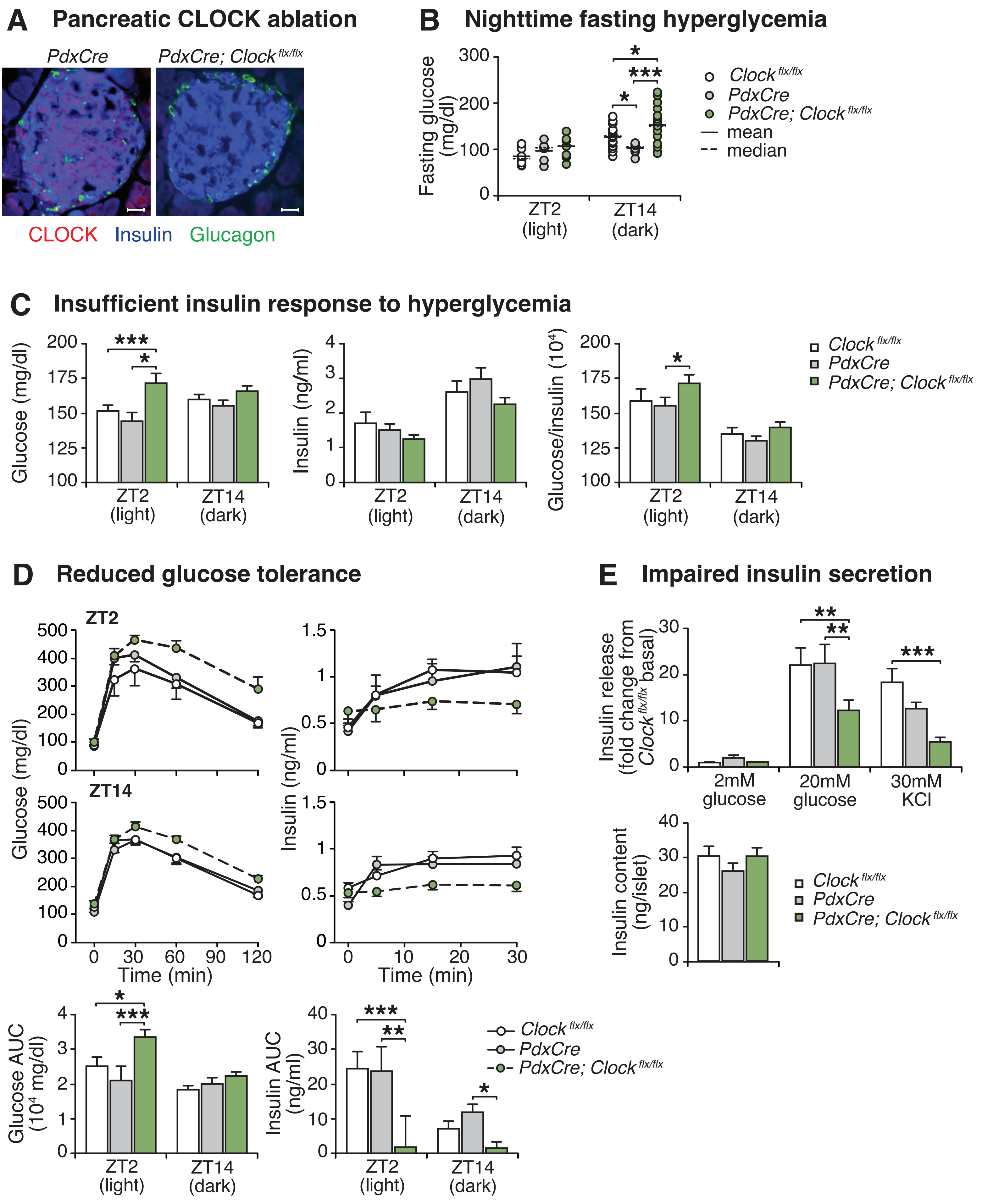
A Pancreatic CLOCK ablation
PdxCre
PdxCre; Clock flx/flx
CLOCK Insulin Glucagon
B Nighttime fasting hyperglycemia
Fasting glucose (mg/dl)
ZT2 (light)
ZT14 (dark)
Clock flx/flx
PdxCre
PdxCre; Clock flx/flx
mean
median
C Insufficient insulin response to hyperglycemia
Glucose (mg/dl)
Insulin (ng/ml)
Glucose/insulin (10⁴)
D Reduced glucose tolerance
ZT2
ZT14
Glucose (mg/dl)
Insulin (ng/ml)
Time (min)
Glucose AUC (10⁴ mg/dl)
Insulin AUC (ng/ml)
E Impaired insulin secretion
Insulin release (fold change from Clock flx/flx basal)
2mM glucose
20mM glucose
30mM KCl
Insulin content (ng/islet)

Figure S3. Ablation of *Clock* gene in pancreatic islets impairs glucose homeostasis similar to pancreas-specific *Bmal1* ablation. (A) Representative immunofluorescent staining of CLOCK (red), insulin (blue), and glucagon (green) in 8 mo old male *PdxCre* and *PdxCre;Clock*$^{flx/flx}$ islets. Scale bars, 25 μm. **(B)** Fasted blood glucose levels in *Clock*$^{flx/flx}$, *PdxCre*, and *PdxCre;Clock*$^{flx/flx}$ mice at ZT2 (n=6-9 per genotype) and ZT14 (n=16-20 per genotype). **(C)** Fed glucose and insulin levels, with corresponding glucose/insulin ratios, in *Clock*$^{flx/flx}$, *PdxCre*, and *PdxCre;Clock*$^{flx/flx}$ mice at ZT2 (16-26 per genotype) and ZT14 (n=16-26 per genotype). **(D)** Glucose and insulin levels at the indicated time points following an intraperitoneal injection of glucose (2 or 3 g/kg body weight, respectively) at ZT2 (n=5-10 per genotype) and ZT14 (n=8-15 per genotype). Background subtracted area under the curve (AUC) (*bottom*) of glucose and insulin responses during glucose tolerance tests shown above. **(E)** Insulin release from islets isolated from 8 mo old pancreas-specific *Clock* knockout, and *PdxCre* and *Clock*$^{flx/flx}$ mice (n=5-7) in response to glucose and KCl. Insulin content in islets isolated from pancreas-specific *Clock* knockout and *PdxCre* and *Clock*$^{flx/flx}$ control mice (n=10-11). Data were analyzed by two-way ANOVA and with repeated measure when appropriate. Tukey tests were used for indicated multiple comparisons. All values represent mean ± SEM. *p<0.05, **p<0.01, ***p<0.001.

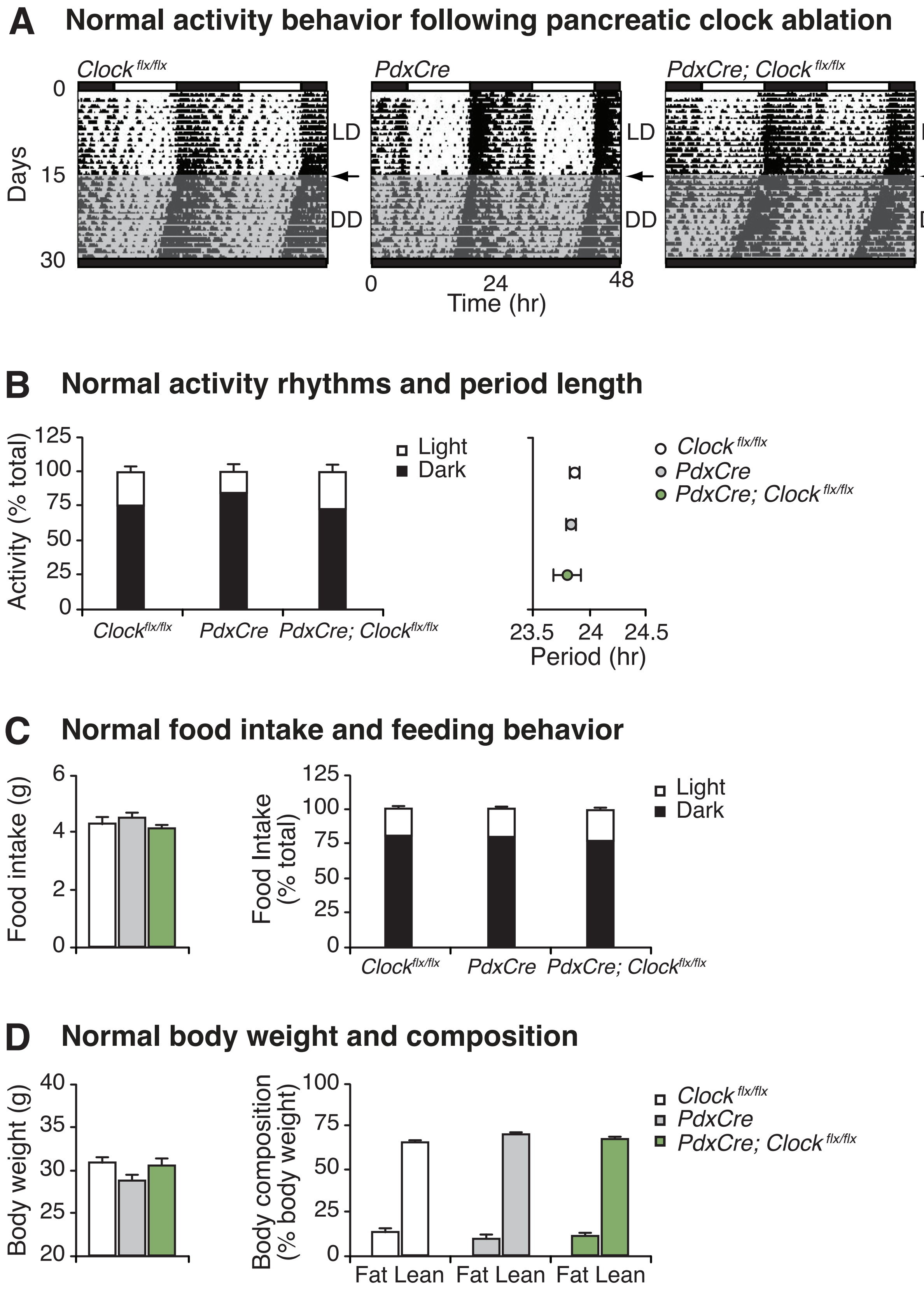
A Normal activity behavior following pancreatic clock ablation
Clock flx/flx
PdxCre
PdxCre; Clock flx/flx
Days
0
15
30
LD
DD
0
24
48
Time (hr)
B Normal activity rhythms and period length
Activity (% total)
125
100
75
50
25
0
Light
Dark
Clockflx/flx
PdxCre
PdxCre; Clockflx/flx
23.5
24
24.5
Period (hr)
C Normal food intake and feeding behavior
Food intake (g)
6
4
2
0
Food Intake (% total)
Light
Dark
D Normal body weight and composition
Body weight (g)
40
35
30
25
20
Body composition (% body weight)
100
75
50
25
0
Fat Lean Fat Lean Fat Lean

Figure S4. Pancreatic *Clock* ablation does not influence activity, feeding rhythms, or weight. **(A)** Actograms showing locomotor activity over a 30 day period in representative 2-4 mo old male $Clock^{flx/flx}$, *PdxCre*, and $PdxCre;Clock^{flx/flx}$ mice. Arrow denotes switch from 12:12 LD to constant darkness (DD). **(B)** Diurnal rhythm of locomotor activity and period length in DD, calculated using Chi-square periodogram for days 18-23 (n=3 per genotype). **(C)** Total daily food intake and percentage of feeding during either the light or dark period (n=8 per genotype). **(D)** Body weight and composition in pancreas-specific *Clock* knockout mice (n=15) and littermate controls (n=17). Data were analyzed by Student's *t*-test. All values represent mean ± SEM.

Figure S5

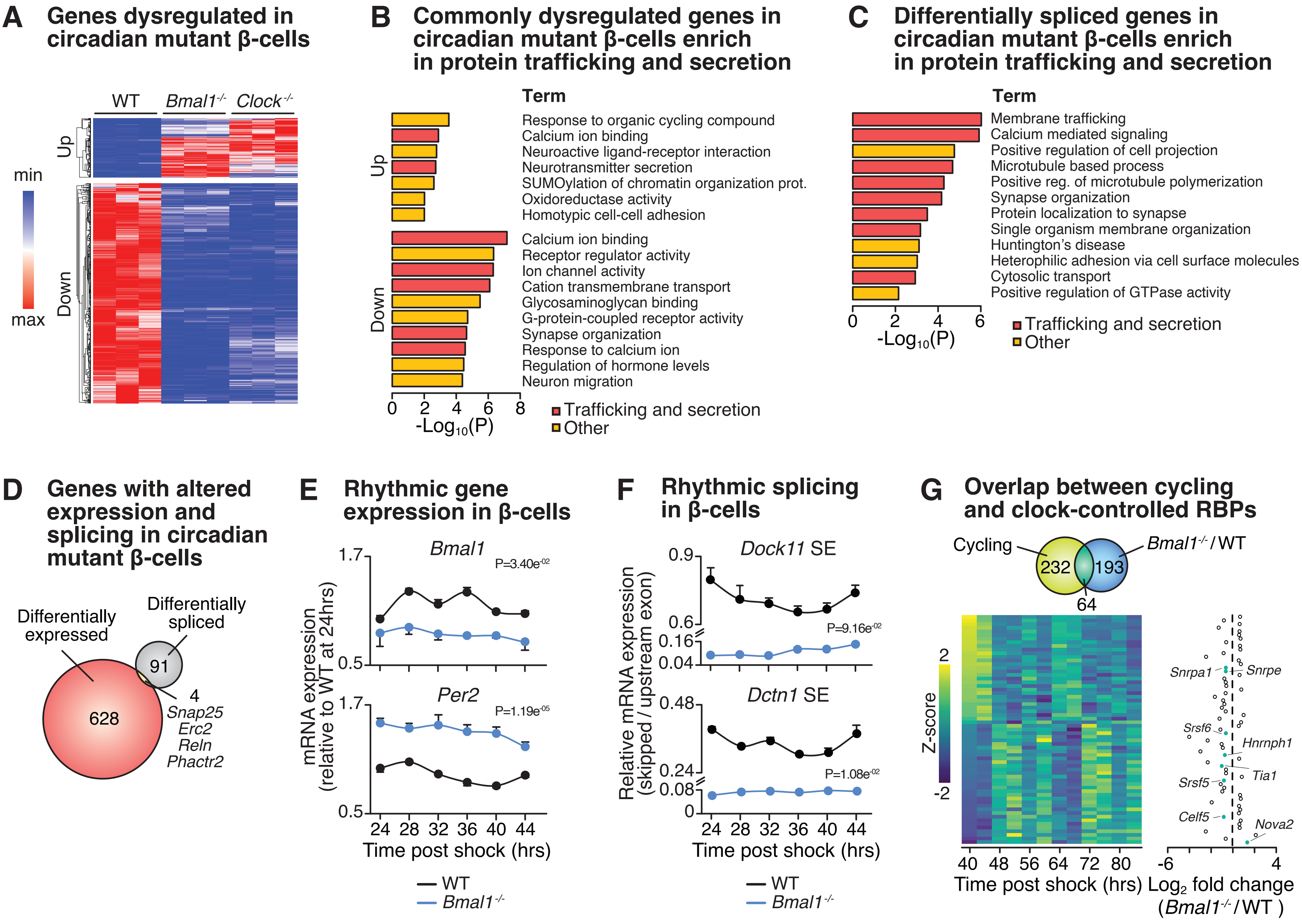

A Genes dysregulated in circadian mutant β-cells
WT
Bmal1-/-
Clock-/-
Up
Down
min
max
B Commonly dysregulated genes in circadian mutant β-cells enrich in protein trafficking and secretion
Term
Up
Response to organic cycling compound
Calcium ion binding
Neuroactive ligand-receptor interaction
Neurotransmitter secretion
SUMOylation of chromatin organization prot.
Oxidoreductase activity
Homotypic cell-cell adhesion
Down
Calcium ion binding
Receptor regulator activity
Ion channel activity
Cation transmembrane transport
Glycosaminoglycan binding
G-protein-coupled receptor activity
Synapse organization
Response to calcium ion
Regulation of hormone levels
Neuron migration
0 2 4 6 8
-Log10(P)
Trafficking and secretion
Other
C Differentially spliced genes in circadian mutant β-cells enrich in protein trafficking and secretion
Term
Membrane trafficking
Calcium mediated signaling
Positive regulation of cell projection
Microtubule based process
Positive reg. of microtubule polymerization
Synapse organization
Protein localization to synapse
Single organism membrane organization
Huntington's disease
Heterophilic adhesion via cell surface molecules
Cytosolic transport
Positive regulation of GTPase activity
0 2 4 6
-Log10(P)
Trafficking and secretion
Other
D Genes with altered expression and splicing in circadian mutant β-cells
Differentially expressed
Differentially spliced
628
91
4
Snap25
Erc2
Reln
Phactr2
E Rhythmic gene expression in β-cells
Bmal1
P=3.40e-02
Per2
P=1.19e-05
1.7
0.5
mRNA expression (relative to WT at 24hrs)
24 28 32 36 40 44
Time post shock (hrs)
WT
Bmal1-/-
F Rhythmic splicing in β-cells
Dock11 SE
P=9.16e-02
0.9
0.6
0.16
0.04
Dctn1 SE
P=1.08e-02
0.48
0.24
0.08
0
Relative mRNA expression (skipped / upstream exon)
24 28 32 36 40 44
Time post shock (hrs)
WT
Bmal1-/-
G Overlap between cycling and clock-controlled RBPs
Cycling
Bmal1-/- / WT
232
193
64
Z-score
2
-2
Snrpa1
Snrpe
Srsf6
Hnrnph1
Tia1
Srsf5
Celf5
Nova2
40 48 56 64 72 80
Time post shock (hrs)
-6 4
Log2 fold change (Bmal1-/- / WT)

Figure S5. Comparison of differentially-expressed and -spliced RNAs in islets and β-cell lines. **(A)** Heatmap of "up" and "down"-regulated differentially expressed genes in both *Bmal1*$^{-/-}$ and *Clock*$^{-/-}$ β-cell lines compared to controls. **(B)** Pathway analyses of gene networks commonly dysregulated in *Bmal1*$^{-/-}$ and *Clock*$^{-/-}$ β-cell lines, revealing enrichment in pathways related to protein trafficking and secretion. (C) Pathway analyses reveal enrichment in trafficking and exocytosis-related genes that are differentially spliced in *Bmal1*$^{-/-}$ and *Clock*$^{-/-}$ β-cell lines. **(D)** Venn diagram showing little overlap between differentially-expressed and differentially-spliced genes in circadian mutant β-cell lines. (**E**) Rhythmic expression of core circadian genes *Bmal1* and *Per2* in forskolin-shocked WT and *Bmal1*$^{-/-}$ β-cell lines as assessed by qPCR. Shown P-value obtained from JTK_CYCLE for WT cells (n=3/timepoint). **(F)** Rhythmic splicing events identified via qPCR in forskolin-synchronized WT and *Bmal1*$^{-/-}$ β-cell lines (n=3/timepoint). Skipped exon expression normalized to neighboring (non-spliced) exon, and evaluation for rhythmicity performed by JTK_CYCLE (adjusted P-value shown). **(G)** Heatmap showing mean expression patterns (z-score) (*left*) and histogram showing $\log_2$ fold change in expression (*right*) in *Bmal1*$^{-/-}$ vs WT for RBPs that are both rhythmic in synchronized islets and differentially expressed in *Bmal1*$^{-/-}$ β-cells.

Figure S6

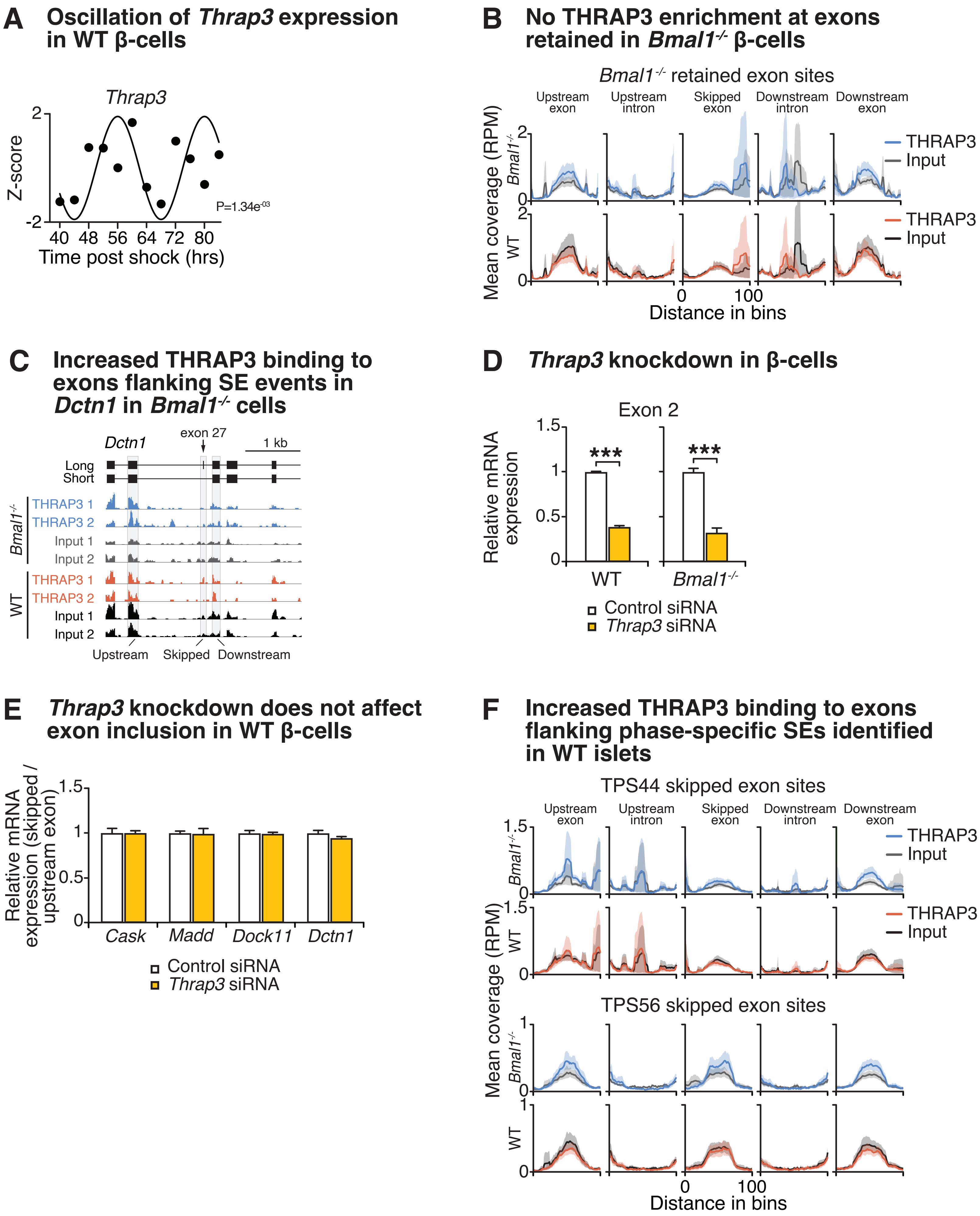
A
Oscillation of Thrap3 expression in WT β-cells
Thrap3
Z-score
P=1.34e-03
Time post shock (hrs)
40 48 56 64 72 80
2
-2
B
No THRAP3 enrichment at exons retained in Bmal1-/- β-cells
Bmal1-/- retained exon sites
Upstream exon
Upstream intron
Skipped exon
Downstream intron
Downstream exon
Mean coverage (RPM)
Bmal1-/-
WT
THRAP3
Input
Distance in bins
0 100
C
Increased THRAP3 binding to exons flanking SE events in Dctn1 in Bmal1-/- cells
Dctn1
exon 27
1 kb
Long
Short
THRAP3 1
THRAP3 2
Input 1
Input 2
Upstream
Skipped
Downstream
D
Thrap3 knockdown in β-cells
Exon 2
Relative mRNA expression
1.5
1
0.5
0
***
WT
Bmal1-/-
Control siRNA
Thrap3 siRNA
E
Thrap3 knockdown does not affect exon inclusion in WT β-cells
Relative mRNA expression (skipped / upstream exon)
Cask
Madd
Dock11
Dctn1
F
Increased THRAP3 binding to exons flanking phase-specific SEs identified in WT islets
TPS44 skipped exon sites
TPS56 skipped exon sites

Figure S6. THRAP3 interacts with clock-dependent rhythmically-spliced RNAs. (A) Rhythmic *Thrap3* expression in forskolin-shocked WT islets cells. Data is shown as mean z-scored RNA-seq expression data from (Perelis et al. 2015). **(B)** Normalized THRAP3 sequencing reads (reads per million) in Beta-TC6 plotted along indicated regions surrounding exons that are retained in *Bmal1*$^{-/-}$ cells compared to WT indicating no enrichment in these regions as mean IP traces are similar to input RNA traces. **(C)** UCSC genome browser tracks showing reads from indicated THRAP3 IP and input samples along regions within the *Dctn1* Refseq transcript. Skipped exon identified by rMATS and flanking exons are shaded in gray and track heights for each sample are equal for all rows. **(D)** Decreased mRNA levels of *Thrap3* in WT and *Bmal1*$^{-/-}$ Beta-TC6 cells after siRNA treatment assessed by qPCR (n=3). **(E)** *Thrap3* siRNA knockdown does not affect exon inclusion in WT Beta-TC6 cells, assessed by qPCR (n=3). **(F)** Normalized THRAP3 sequencing reads (reads per million) in Beta-TC6 plotted along indicated regions surrounding exons that are skipped in islet cells at indicated time points. Solid line represents mean for two replicates and shading represents 95% confidence interval between samples. Increased reads in IP samples vs input indicate occupancy of THRAP3 on flanking exons for these regions in *Bmal1*$^{-/-}$ cells.

Figure S7

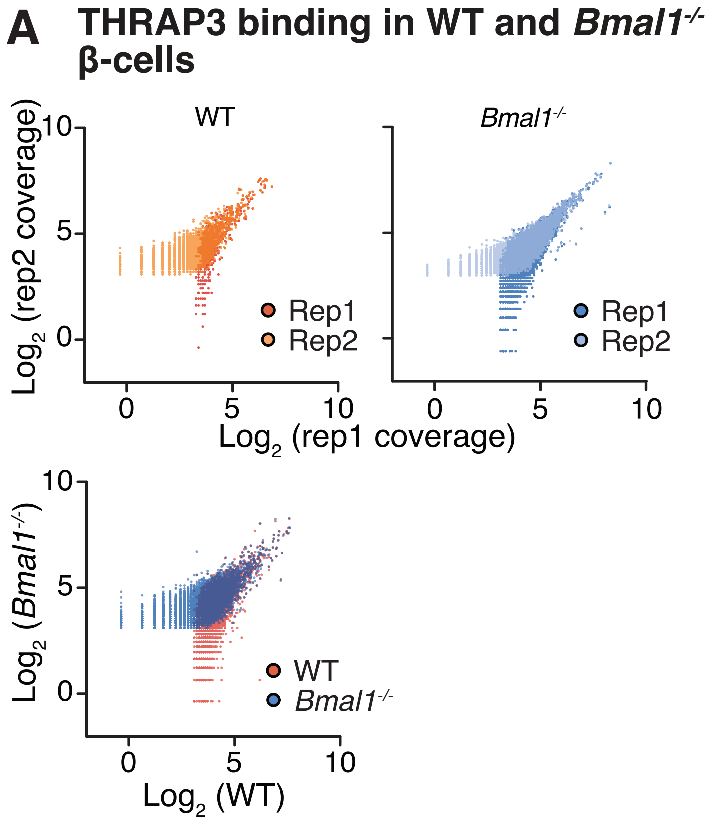
A
THRAP3 binding in WT and Bmal1-/- β-cells
WT
Bmal1-/-
Log2 (rep2 coverage)
Log2 (rep1 coverage)
Rep1
Rep2
10
5
0
Log2 (Bmal1-/-)
Log2 (WT)
WT
Bmal1-/-

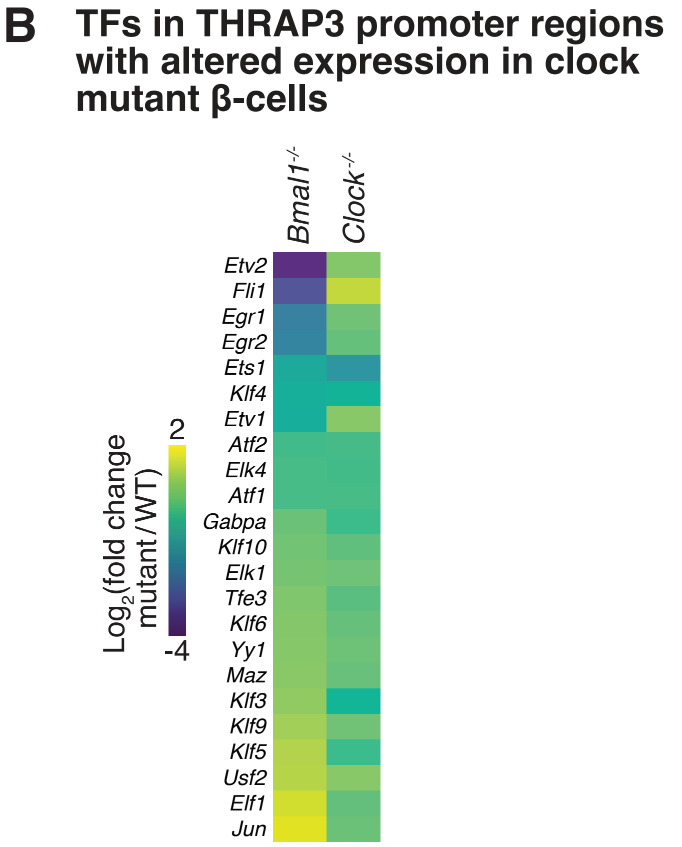
B
TFs in THRAP3 promoter regions with altered expression in clock mutant β-cells
Bmal1-/-
Clock-/-
Log2(fold change mutant/WT)
2
-4
Etv2
Fli1
Egr1
Egr2
Ets1
Klf4
Etv1
Atf2
Elk4
Atf1
Gabpa
Klf10
Elk1
Tfe3
Klf6
Yy1
Maz
Klf3
Klf9
Klf5
Usf2
Elf1
Jun

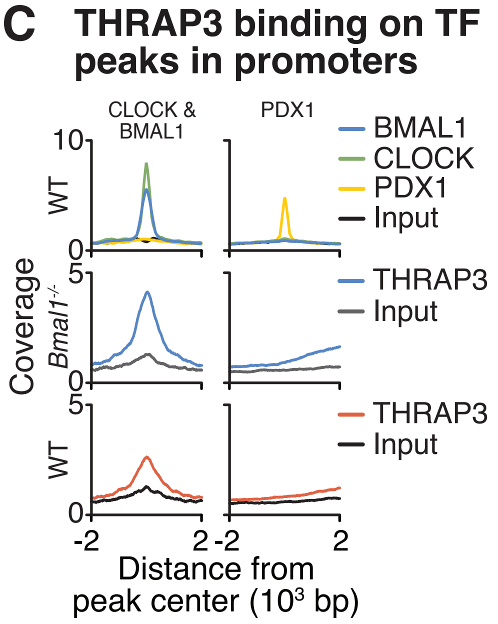
C
THRAP3 binding on TF peaks in promoters
CLOCK & BMAL1
PDX1
BMAL1
CLOCK
PDX1
Input
THRAP3
Input
THRAP3
Input
WT
Bmal1-/-
WT
Coverage
10
5
0
-2
2
Distance from peak center (10³ bp)

Figure S7. THRAP3 ChIP-sequencing in BetaTC6 cells. (**A**) Scatterplots showing normalized sequencing reads log_2 (reads per million) counted across 500 bp windows surrounding indicated sets of peaks where reads from replicate 1 are plotted on the x-axis and replicate 2 are plotted on the y-axis (*top*). Comparison between replicates with the largest numbers of peaks (WT replicate 2, *Bmal1*$^{-/-}$ replicate 1), where reads from WT are plotted on the x-axis and *Bmal1*$^{-/-}$ are plotted on the y-axis (*bottom*). **(B**) Heatmap showing expression of TFs with known motifs enriched in THRAP3 peaks localized to promoters or in promoters of alternatively spliced genes with altered expression in circadian mutant β-cell lines. **(C**) Histograms showing normalized ChIP-seq signal (reads per million) for indicated transcription factors and THRAP3 binding on CLOCK/BMAL1 and PDX1 peaks located in promoters.

Figure S8

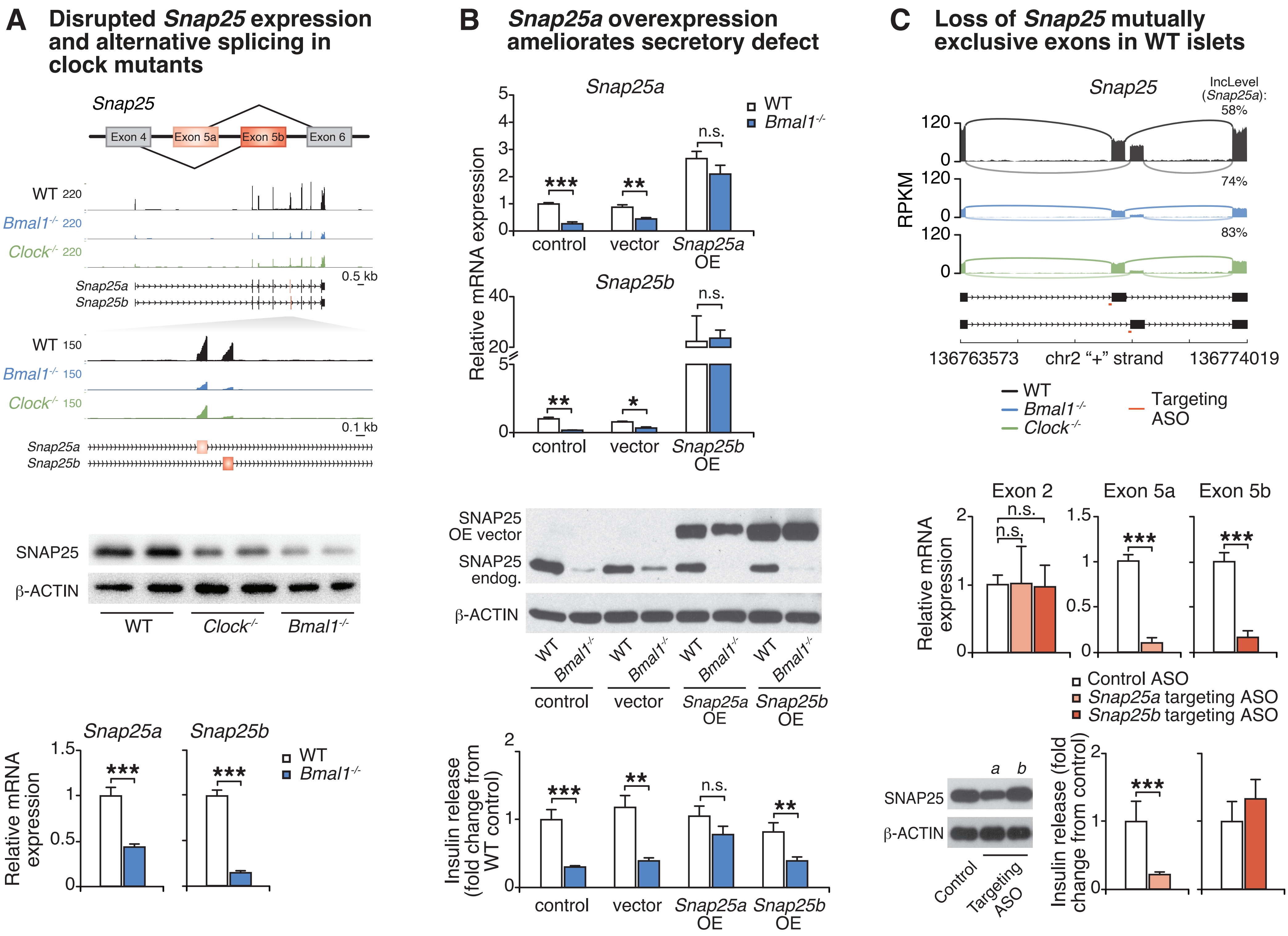

A Disrupted Snap25 expression and alternative splicing in clock mutants
Snap25
Exon 4
Exon 5a
Exon 5b
Exon 6
WT 220
Bmal1-/- 220
Clock-/- 220
0.5 kb
Snap25a
Snap25b
WT 150
Bmal1-/- 150
Clock-/- 150
0.1 kb
SNAP25
β-ACTIN
WT
Clock-/-
Bmal1-/-
Relative mRNA expression
WT
Bmal1-/-
B Snap25a overexpression ameliorates secretory defect
Relative mRNA expression
control
vector
Snap25a OE
Snap25b OE
n.s.
SNAP25 OE vector
SNAP25 endog.
Insulin release (fold change from WT control)
C Loss of Snap25 mutually exclusive exons in WT islets
IncLevel (Snap25a):
58%
74%
83%
RPKM
136763573
chr2 "+" strand
136774019
Targeting ASO
Exon 2
Exon 5a
Exon 5b
Control ASO
Snap25a targeting ASO
Snap25b targeting ASO
Control
Targeting ASO
Insulin release (fold change from control)

Figure S8. Reduced expression of the *Snap25a* isoform in circadian mutant β-cells contributes to impaired insulin secretion. (A) Browser tracks showing disrupted expression and alternative splicing of *Snap25* in *Bmal1*$^{-/-}$ and *Clock*$^{-/-}$ Beta-TC6 cells (*top*). Decreased SNAP25 protein expression by Western blot in *Clock*$^{-/-}$ and *Bmal1*$^{-/-}$ Beta-TC6 cells compared to WT controls (*middle*). Quantitative real-time PCR to assess expression of *Snap25* mutually-exclusive exons 5a and 5b in *Bmal1*$^{-/-}$ Beta-TC6 cells (n=6 per genotype) (*bottom*). **(B)** Overexpression of empty vector (n=3), *Snap25a* (n=4), and *Snap25b* (n=5) isoforms in WT and *Bmal1*$^{-/-}$ Beta-TC6 cell lines, compared to control cells (n=3), as assessed by qPCR (*top*). Overexpression of SNAP25A or B isoforms or empty vector by Western blot in WT and *Bmal1*$^{-/-}$ Beta-TC6 cell lines (*middle*). Insulin release in control (n=11-12 samples), empty vector (n=4), *Snap25a*- (n=8-11), or *Snap25b*- (n=7-9) overexpressing pseudoislets (*bottom*). **(C)** Sashimi plots of the differentially spliced *Snap25* gene in *Bmal1*$^{-/-}$ and *Clock*$^{-/-}$ β-cell lines (*top*). Quantitative real-time PCR to assess expression of non-targeted exon (exon 2) of *Snap25* following control (n=5) and targeting ASO treatment (n=3-5) in WT islets (*middle left*). Expression of *Snap25* exon 5a or 5b by qPCR (*middle right*) following control (n=5 mice) or targeting ASO (n=6 mice) treatment in WT islets. SNAP25 protein levels after ASO treatment in WT Beta-TC6 cell lines as assessed by Western blot (*bottom left*). Insulin secretion (*bottom right*) following control (n=8 mice) or targeting ASO (n=15 mice) treatment in WT islets. Comparisons were made by Student's *t*-test with P-value multiple comparison corrections using the Holm-Sidak method. All values represent mean ± SEM. *p<0.05, **p<0.01, ***p<0.001.

Figure S9

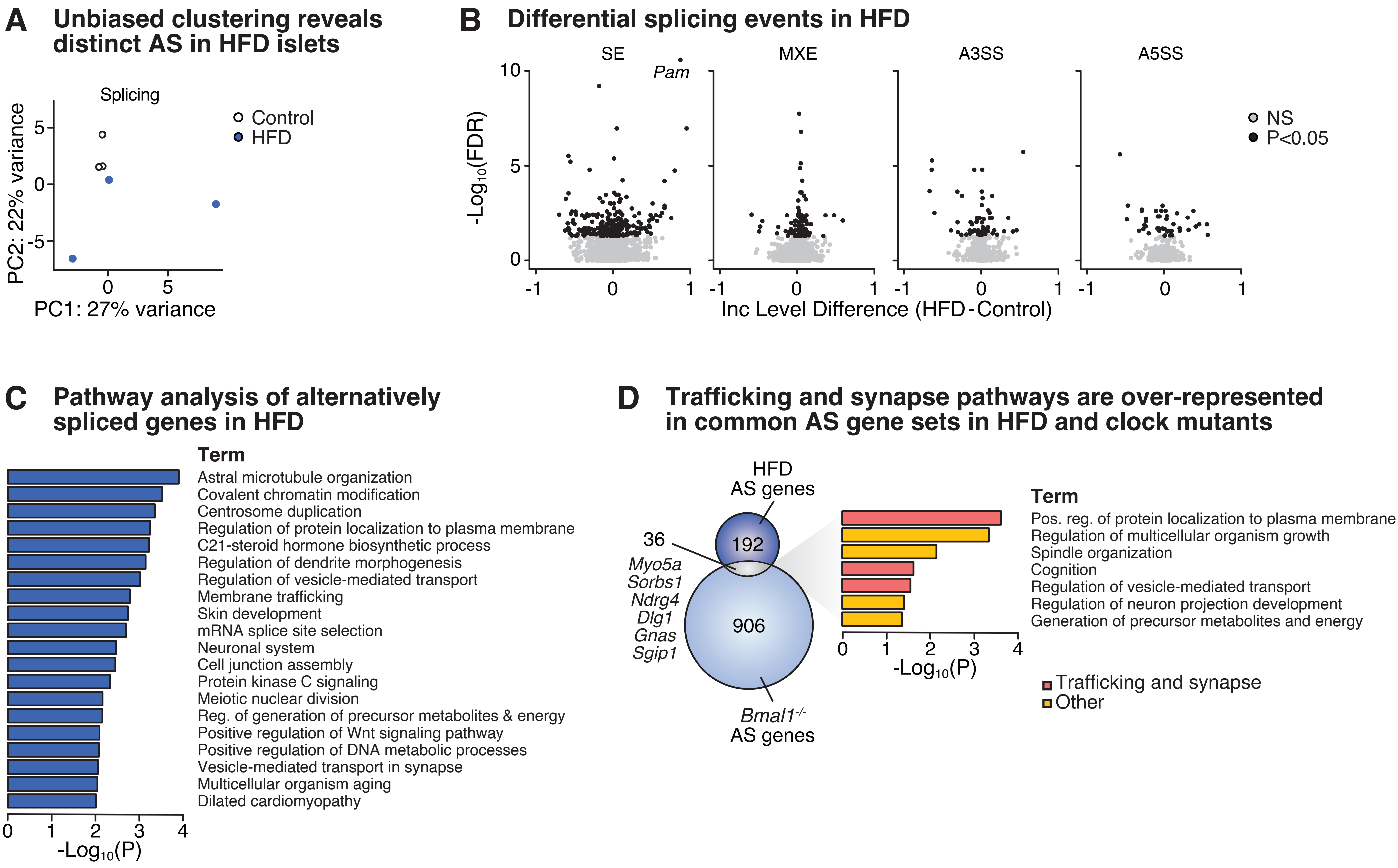
A Unbiased clustering reveals distinct AS in HFD islets
Splicing
Control
HFD
PC2: 22% variance
PC1: 27% variance
5
0
-5
0
5
B Differential splicing events in HFD
SE
MXE
A3SS
A5SS
Pam
-Log10(FDR)
10
5
0
-1
0
1
Inc Level Difference (HFD - Control)
NS
P<0.05
C Pathway analysis of alternatively spliced genes in HFD
Term
Astral microtubule organization
Covalent chromatin modification
Centrosome duplication
Regulation of protein localization to plasma membrane
C21-steroid hormone biosynthetic process
Regulation of dendrite morphogenesis
Regulation of vesicle-mediated transport
Membrane trafficking
Skin development
mRNA splice site selection
Neuronal system
Cell junction assembly
Protein kinase C signaling
Meiotic nuclear division
Reg. of generation of precursor metabolites & energy
Positive regulation of Wnt signaling pathway
Positive regulation of DNA metabolic processes
Vesicle-mediated transport in synapse
Multicellular organism aging
Dilated cardiomyopathy
0
1
2
3
4
-Log10(P)
D Trafficking and synapse pathways are over-represented in common AS gene sets in HFD and clock mutants
HFD AS genes
192
36
Myo5a
Sorbs1
Ndrg4
Dlg1
Gnas
Sgip1
906
Bmal1-/- AS genes
Term
Pos. reg. of protein localization to plasma membrane
Regulation of multicellular organism growth
Spindle organization
Cognition
Regulation of vesicle-mediated transport
Regulation of neuron projection development
Generation of precursor metabolites and energy
0
1
2
3
4
-Log10(P)
Trafficking and synapse
Other

Figure S9. High fat feeding disrupts alternative splicing of trafficking genes, similar to circadian mutant islets. (A) Unbiased clustering using principle component analysis (PCA) of splicing frequencies in publicly available RNA-seq data from HFD (n=3) and control-fed (n=3) mouse islets reveals distinct landscape of AS events with respect to diet along PC2. (**B**) HFD results in 229 differential AS events among 200 unique genes, including several genes implicated in β-cell function (i.e. *Pam*). **(C)** Pathway analyses of alternatively spliced genes in islets isolated from HFD-fed mice. **(D)** Venn diagram showing overlap between genes alternatively spliced in islets from HFD-fed mice and from *Bmal1*$^{-/-}$ β-cell lines. Pathway analyses of the common set of alternatively-spliced gene sets in islets isolated from HFD-fed mice and *Bmal1*$^{-/-}$ β-cells reveal enrichment in vesicle trafficking and synapse related pathways.

Table S1. Rhythmic alternatively-spliced genes in WT islets

Table S2. RNA-binding proteins with rhythmic expression in WT islets

Table S3. Genes with altered expression in *Bmal1*$^{-/-}$ and *Clock*$^{-/-}$ vs wild-type β-cells

Table S4. Genes with altered splicing in *Bmal1*$^{-/-}$ and *Clock*$^{-/-}$ vs wild-type β-cells

Table S5. Genes with altered splicing in HFD vs control islets

Table S6. qPCR primer and ASO sequences
